# Conservation of giant genome structure in Brazilian and Chilean species of the genus *Alstroemeria* L. (Alstroemeriaceae), despite dynamism in satellite repeats

**DOI:** 10.1007/s00425-026-04933-z

**Published:** 2026-02-06

**Authors:** Jéssica Nascimento, Mariela Sader, Oscar Toro-Núñez, Carlos Baeza, Yennifer Mata-Sucre, Leonardo Félix, Andrea Pedrosa-Harand

**Affiliations:** 1 Laboratory of Plant Cytogenetics and Evolution, Department of Botany, Biosciences Centre, Federal University of Pernambuco, Recife, Brazil; 2 Multidisciplinary Institute of Plant Biology (National Council for Scientific and Technical Research - National University of Córdoba), Córdoba, Argentina; 3 Department of Botany, Faculty of Natural and Oceanographic Sciences, Universidad de Concepción, Concepción, Chile; 4 Institute of Genetics, Reproduction and Development (iGReD), CNRS, Inserm, Université Clermont Auvergne, 63000 Clermont-Ferrand, France; 5 Laboratory of Plant Cytogenetics and Systematics, Department of Biosciences, Federal University of Paraíba, Areia, PB Brazil

**Keywords:** Genome skimming, Repeatome, Retrotransposons, Satellite DNA, Obese genomes

## Abstract

**Main conclusions:**

Giant genomes in *Alstroemeria* have maintained structural conservation for ~18.4 million years, whereas satellite DNA amplification and elimination constitutes the main dynamic force underlying heterochromatin diversification and longitudinal chromosomal differentiation.

**Abstract:**

Repetitive sequences are major components of plant genomes and play key roles in genome size evolution and structural variation. *Alstroemeria* L. is a genus of monocotyledonous plants with giant genomes (1C ≈ 25 Gb), native to the Americas and distributed into two distinct lineages: the Brazilian/Argentinean clade and the Chilean grade. Despite their ancient separation and differences in heterochromatin distribution, all species share a highly conserved chromosome number (2*n* = 16), with only minor variation in chromosome morphology. Here, we characterized the repetitive DNA fraction of six Chilean species, one Argentinean species, and two Brazilian species, and mapped the most abundant repeats on representative chromosomes from each lineage. LTR Ty3/gypsy Tekay retrotransposons were the predominant repetitive elements, accounting for 30.63–39.91% of the genome across all analyzed species and largely explaining genome size variation. Notably, despite their giant size, *Alstroemeria* genomes exhibited a relatively low overall proportion of repetitive DNA (up to ~68%), consistent with slow repeat removal and the accumulation of degraded sequences, as predicted for genomes of this size. Satellite DNA represented 0.23–3.42% of the genome, with most satellite families shared between the Brazilian and Chilean species. Nevertheless, despite the divergence of the Brazilian lineage approximately 9.2 million years ago, marked differences in satellite abundance and chromosomal distribution were observed. Our results indicate that giant genome evolution in *Alstroemeria* is characterized by long-term conservation of karyotype structure and transposable element composition, whereas satellite DNA constitutes a key dynamic component associated with heterochromatin diversification and longitudinal chromosomal differentiation between the Chilean and Brazilian lineages.

**Supplementary Information:**

The online version contains supplementary material available at 10.1007/s00425-026-04933-z.

## Introduction

Angiosperms represent the most diverse group of plants, occupying a wide range of environments. This remarkable diversity is reflected not only in their morphology—through adaptations to different habitats—but also in their genomes, including variation in chromosome number, structure, genome size, and composition. Several factors contribute to genome differentiation, even among closely related species. In plants, the two main drivers are polyploidy (WGD—whole genome duplication; Wood et al. 2009; Soltis and Soltis 2020) and the expansion or contraction of DNA sequences, which cause genomic “fattening” or “shrinking” (Kelly et al. 2015; Macas et al. 2015; Sader et al. 2021).

Plant genomes consist of various types of sequences, which may occur as single-copy, low-copy, or highly repeated sequences (Novák et al. 2020a; Thakur et al. 2021). The repetitive DNA fraction is often responsible for genomic differentiation due to its rapid turnover and homogenization, potentially giving rise to species-specific sequences (Kelly et al. 2015; McCann et al. 2020). These sequences are commonly located in heterochromatin, a gene-poor region typically found in pericentromeric and subtelomeric regions (Guerra 1988, 2000; Saksouk et al. 2015; Thakur et al. 2021). However, heterochromatin is highly dynamic, and its composition and chromosomal distribution can vary significantly among species (Garrido-Ramos 2015, 2017, 2021).

Repetitive DNA is generally categorized into two main types: dispersed elements, which are scattered throughout the genome, and tandemly repeated sequences, which form arrays or clusters. The dispersed sequences are mostly transposable elements (TEs), which can move within the genome without the need for sequence homology, making them particularly successful in spreading across the host genome (Feschotte et al. 2002). TEs are classified into Class I (retrotransposons) and Class II elements (DNA transposons), based on their transposition mechanisms (Muñoz-López and García-Pérez 2010; Bourque et al. 2018; Neumann et al. 2019). Retrotransposons are the most abundant in plant genomes and are typically grouped into LTR (long terminal repeat) elements and non-LTR elements, such as LINEs. LTR retrotransposons are further divided into the Ty1/copia and Ty3/gypsy superfamilies, which constitute a significant proportion of the repeatome in many plant species. This pattern has been observed in *Zea mays*, where repeats account for up to 85% of the genome (Schnable et al. 2009), as well as in *Melampodium glabribracteatum* (~60%) (McCann et al. 2020) and *Passiflora quadrangularis* (66.98%) (Sader et al. 2021), highlighting their major role as drivers of genome evolution (Pellicer et al. 2018).

Although representing a smaller fraction of the genome compared to transposable elements (TEs), tandemly repeated DNAs include, for example, both ribosomal DNA (rDNA), which is essential for cellular function, and satellite DNAs (satDNAs), which play important structural and evolutionary roles, primarily contributing to chromatin organization and lineage-specific genome evolution patterns. SatDNAs are arranged in tandem head-to-tail arrays and often constitute the primary structural component of heterochromatic blocks visible by cytogenetic staining (Rao et al. 2010), along with other types of repetitive sequences. These sequences are highly diverse in both copy number and sequence and their rapid evolution and homogenization may lead to species-specific patterns or conservation among related species (Hemleben et al. 2007; Garrido-Ramos 2017, 2021; Ribeiro et al. 2020). Because they are major components of heterochromatin in many plants, satDNAs are useful markers in chromosomal evolution studies, particularly in taxa with limited variation in chromosome morphology.

The genus *Alstroemeria*, the second most diverse in the monocot family Alstroemeriaceae (Liliales; APG IV, 2020), comprises approximately 80 species primarily distributed across southern South America (Andean region) and eastern Brazil (Finot et al. 2018a). These herbaceous species exhibit remarkable variability in flower morphology and color, making them valuable as ornamentals and among the most cultivated cut and potted flowers worldwide (Girardi et al. 2017; Dhiman and Kashyap 2022). Although found in diverse habitats—ranging from deserts, savannas, and forests, to montane regions, and high-altitude areas (Assis 2002; Finot et al. 2018b)—species are mainly concentrated in Brazil and Chile (Baeza et al. 2012). The Brazilian species form a clade, the so-called Brazilian *Alstroemeria* clade, within the Chilean species, which form a paraphyletic grade (Chacón et al. 2012).

Despite diversifying around 18.4 million years ago, and with the Brazilian clade emerging ~9.2 million years ago (Chacón et al. 2012), cytogenetic data show a stable chromosome number (2*n* = 16) across the genus (Chilean Plants Cytogenetics Database, https://chileanpcd.com). Nonetheless, the species exhibit pronounced karyotypic asymmetry and large genomes (Sanso 1996, 2002; Sanso and Hunziker 1998; Baeza et al. 2010, 2016; Ribeiro et al. 2021a; Buitendijk et al. 1997). A major karyotypic difference lies in heterochromatin patterns. The Chilean species typically show prominent interstitial and telomeric heterochromatic bands, estimated, by chromosome measurements, to comprise ~16.8% of the chromosome complement (CC) in species with large genomes (ca. 1 C = 27.04 Gbp) (Buitendijk and Ramanna 1996), and reduced bands (~2.0% CC) in species with smaller genomes (ca. 1 C = 20.34 Gbp) (Buitendijk and Ramanna 1996). In contrast, the Brazilian species exhibit consistently small heterochromatic bands (<1.0% CC), regardless of genome size (ca. 1 C = 24.45 Gbp; Buitendijk and Ramanna 1996; Buitendijk et al. 1997). The same pattern was confirmed using CMA/DAPI staining (Zanela 2009; Ribeiro et al. 2021a), even in the Brazilian species with large genomes (up to 1 C = 27.58 Gbp), suggesting that heterochromatin in the Brazilian species may be more dispersed than in their Chilean counterparts and that their genomes may have followed distinct evolutionary trajectories (Buitendijk and Ramanna 1996).

To date, the repeatome of Alstroemeriaceae remains poorly characterized, with only two species analyzed in detail: the Brazilian *Alstroemeria longistaminea* and *Bomarea edulis* (Ribeiro et al. 2021a, b; Nascimento et al. 2025a). In *A. longistaminea*, a high abundance of retrotransposons and a relatively low proportion of satellite DNAs (satDNAs) were observed. However, the identified satDNAs displayed considerable diversity in terms of abundance, monomer length, and chromosomal localization. Of the 16 satDNA families detected, 10 were mapped using FISH: three localized to heterochromatic bands, two formed multiple clusters along euchromatic regions in a G-band-like pattern, and five were dispersed in euchromatin with occasional heterochromatic enrichment (Ribeiro et al. 2021b). In this context, low-coverage genome sequencing proves to be a valuable tool for characterizing the repetitive DNA fraction, particularly for analyzing and identifying key satDNAs potentially involved in the longitudinal chromosomal differentiation observed among species.

In this study, we aimed to investigate the evolution and organization of heterochromatin in the Chilean and Brazilian species of *Alstroemeria*. We characterized the repetitive DNA fraction of six Chilean, one Argentinean, and two additional Brazilian species, and mapped the most abundant repeats along the chromosomes of representative species from both groups.

## Materials and methods

### Plant materials

One individual of *Alstroemeria urubiciensis* Herb., collected in Urubici, Santa Catarina, Brazil, and one individual of *A. monticola* Mart. ex Schult. and Schult. f., collected in Jaguarari, Bahia, Brazil, were cultivated in the experimental garden of the Laboratory of Plant Cytogenetics and Evolution at the Federal University of Pernambuco (UFPE), Recife, Pernambuco. Voucher specimens were deposited in the EAN herbarium (Prof. Jayme Coelho de Moraes, Federal University of Paraíba—UFPB, Areia, PB, Brazil). For the Brazilian species *A. longistaminea* Mart. (EAN—LPF 15942), 150 bp HiSeq 4000 (Illumina) reads were obtained from a previous study (Ribeiro et al. 2021b), in collaboration with Dr. Tiago Ribeiro (Federal University of Mato Grosso, Brazil). The Brazilian/Argentinean species *A. psittacina* Lehm., collected in Córdoba, Argentina, is maintained at the Multidisciplinary Institute of Plant Biology, National University of Córdoba. The Chilean species *A. exerens* Meyen, *A. hookeri* Lodd. subsp. *hookeri*, *A. ligtu* L. subsp. *ligtu*, *A. pulchra* Sims subsp. *pulchra*, *A. philippii* Baker subsp. *philippi*, and *A. violacea* Phil. were collected from various localities, and voucher specimens were deposited in the CONC herbarium (University of Concepción, Concepción, Chile). Collection data, voucher information, NCBI sequence accession numbers, and genome sizes are summarized in Table 1 and Supplementary Table 1..

**Table 1:** Collection data and vouchers of *Alstroemeria* species analyzed

Species	Voucher	NCBI accession number	Location
*A. exerens*	Arroyo 29,140	SAMN51256950	Santiago Province, Road to Valle Nevado—Chile
*A. hookeri* subsp. *hookeri*	Toro-Núñez 151	SAMN51256951	Biobío Region, Biological station of the University of Concepción, Concepción Province—Hualpen Sector—Chile
*A. urubiciensis*	LPF 19355	SAMN51256952	Urubici, Santa Catarina—Brasil
*A. ligtu* subsp. ligtu	Toro-Núñez 150	SAMN51256953	Biobío Region, Biological station of the University of Concepción, Concepción Province—Explanada superior sector—Chile
*A. longistaminea*	*	-	*
*A. monticola*	LPF 18804	SAMN51256954	Jaguarari, Bahia—Brasil
*A. philippii* subs *philippii*	Carrasco 104	SAMN51256955	Huasco Province, Atacama Region—Road to Aguada de Tongoy—Chile
*A. psittacina*	MS001CORD	SAMN51256956	Córdoba—Argentina
*A. pulchra* subs *pulchra*	Carrasco 116	SAMN51256957	Valparaíso Province, Valparaíso Region, Ritoque sector—Chile
*A. violacea*	Carrasco 100	SAMN51256958	Copiapó Province, Atacama Region, Quebrada El Leon—Chile

*Ribeiro et al. (2021b)

**Table 2:** Genomic proportions (%) of repetitive sequences identified in species of *Alstroemeria* after individual RepeatExplorer analysis

	*A. exerens*	*A. hookeri*	*A. urubiciensis*	*A. ligtu*	*A. monticola*	*A. philippii*	*A. psittacina*	*A. pulchra*	*A. violacea*
Individual clustering reads	5.133.937	4.422.613	4.069.740	1.575.672	2.823.296	5.414.146	6.112.972	4.684.132	5.450.601
Coverage	–	0.03×	0.02×	0.007×	0.02×	0.04×	0.04×	0.04×	0.03×
Repetitive elements				Genome proportion (%)					
Class I									
LTR -retrotransposons									
Ty1/copia									
Tork	3.22	3.36	3.17	2.94	3.06	3.73	2.86	4.30	3.87
SIRE	0.84	1.44	1.34	1.11	0.77	1.87	0.74	2.6	1.89
Angela	0.74	0.84	1.05	–	1.07	1.01	1.03	0.7	0.88
TAR	0.33	0.37	0.39	0.22	0.30	0.43	0.36	0.32	0.42
Ivana	0.11	0.27	0.02	0.06	0.01	0.35	0.28	0.06	0.36
Ale	0.11	0.31	0.18	0.04	0.18	0.21	0.21	0.21	0.19
Ikeros	0.02	0.02	–	–	–	0.01	–	0.01	0.01
Alesia	–	–	0.01	–	–	–	–	–	–
Ty3/gypsy									
*Chromovirus*									
Tekay	35.74	38.70	34.42	36.94	32.60	39.91	36.65	30.63	33.70
CRM	2.28	2.74	3.6	2.02	3.86	2.99	1.99	2.58	2.78
Galadriel	0.07	0.08	0.04	-	-	0.07	0.02	0.1	0.10
Non-chromovirus									
Retand	3.62	5.85	6.45	5.87	5.29	5.20	5.12	11.03	5.10
Athila	0.71	0.59	0.04	0.40	0.23	0.60	0.04	0.03	0.57
Others LTRs	0.92	2.24	3.69	0.46	3.20	0.85	5.49	3.51	2.25
Non-LTR									
LINE	0.43	0.48	0.60	3.20	0.69	0.48	0.53	0.53	0.59
Pararetrovirus	0.02	0.03	0.03	-	0.04	0.13	0.07	-	0.07
Class II									
CACTA	1.80	2.15	3.20	2.06	2.55	1.92	3.06	1.68	1.92
Mutator	0.61	0.77	0.84	0.25	0.51	0.53	1.27	0.6	0.49
Harbinger	0.33	0.03	0.01	0.01	0.16	0.38	0.39	0.3	0.36
hAT	0.05	0.05	0.08	-	0.05	0.06	0.08	0.05	0.08
SatDNA	2.00	2.65	1.70	3.42	0.73	0.63	0.23	2.43	0.56
rDNA									
5S	0.11	0.44	0.06	0.09	0.05	0.03	0.05	0.03	0.02
35S	0.68	0.58	0.46	0.59	0.28	0.91	0.13	1.14	0.62
Unclassifield repeats	14	3.35	4.20	2.06	6.10	5.21	5.5	4.02	6.82
Total	68.79	67.43	65.60	62.30	61.73	67.60	66.10	66.86	63.65

### Genome size estimation by flow cytometry

Nuclear DNA contents of *A. monticola* and *A. urubiciensis* were estimated by flow cytometry using a CyFlow SL cytometer (Partec, Görlitz, Germany). Nuclei suspensions were prepared from young leaves in WPB buffer (Loureiro et al. 2007), and interphase nuclei were stained with propidium iodide. *Vicia faba* (2C = 26.9 pg; Dolezel et al. 2007) was used as an internal standard. Genome size (2C value) was calculated using the following formula based on three independent replicates performed on different days: (Mean sample peak/Mean standard peak) ×  2 C DNA content of internal standard (pg).

### Genome size estimation by karyotypic comparison

The approximate genome size of *A. violacea* was indirectly estimated by comparing the total length of its haploid karyotype (HKL) to that of *A. longistaminea* (2*n* = 16; HKL = 106.97 μm; Ribeiro et al. 2021a), for which genome size is known (1C = 25.8 Gbp; Nascimento et al. 2025a). A linear relationship was assumed between haploid karyotype length (in micrometers) and genome size (in picograms). Thus, a simple rule-of-three proportion was applied as follows: (Genome size of species Y [pg]/HKL of species Y [μm]) = (Estimated genome size of species X [pg]/HKL of species X [μm]). For the remaining species, genome size values were obtained from the literature (Buitendijk et al. 1997; Nascimento et al. 2025a). Since no chromosomal or genome size data are available for *A. exerens*, sequencing coverage could not be estimated for this species.

### DNA extraction, NGS sequencing and data processing

Genomic DNA from the Brazilian and Argentinean species was extracted from young leaves following the protocol of Weising et al. (2005). DNA from the Chilean species was extracted using the DNeasy Plant Mini Kit (Qiagen). Paired-end 150 bp reads were generated through low-coverage DNBSeq sequencing (BGI Group, Hong Kong, China). Raw reads were quality filtered, retaining only those in which 90% of the bases had a quality score ≥ Q10. Clustering analysis based on sequence similarity was conducted using the RepeatExplorer2 pipeline on the Elixir-CERIT server (https://repeatexplorer-elixir.cerit-sc.cz) (Novák et al. 2013, 2020b).

Two analyses were performed: (1) An individual analysis of each species, based on genome sequencing at the following coverages after automatic sampling of 1 Gb input data each: ~0.007× for *A. ligtu*, 0.02× for *A. monticola* and *A. urubiciensis,* 0.03× for *A. hookeri* and *A. violacea*, and 0.04× for *A. philippii*, *A. psittacina* and *A. pulchra* (see Table 2). For *A. exerens*, it was not possible to estimate genome coverage, as no genome size or chromosomal data are available in the literature. (2) A comparative analysis using sequences from all nine species and *A. longistaminea* (previously analyzed by Ribeiro et al. 2021b). For the second analysis, we use the interlaced reads from each species, first identified with a prefix and then concatenated. The comparative analysis was carried out with a total of 6,085,095 reads with the following coverages after automatic sampling: 622,730 of *A. exerens*, 607,382 of *A. hookeri* (0.004×), 649,280 of *A. longistaminea* (0.004×), 606,124 of *A. philippii* (0.004×), 724,394 of *A. psittacina* (0.004×), 539,346 of *A. pulchra* (0.004×), 608,328 of *A. ligtu* (0.003×), 557,148 of *A. monticola* (0.003×), 558,864 of *A. urubiciensis* (0.003×), and 611,498 of *A. violacea* (0.003×)*.* Genome coverage for each species was calculated through the formula *(r* × *l)/g*, where *r* corresponds to the number of reads used in analysis, *l* corresponds to the length of the reads and *g* the size of the haploid genome in bp (Supplementary Table 1).

### Clustering analysis and characterization of the repetitive fraction

Clusters with a minimum genomic abundance of 0.01% were automatically annotated following the classification system of Neumann et al. (2019) and manually curated to identify the most abundant repetitive families. A custom satellite DNA database specific to *Alstroemeria* was incorporated into both analyses. Unclassified clusters were further analyzed using BLASTN similarity searches against non-redundant protein sequences in public databases (https://blast.ncbi.nlm.nih.gov/Blast.cgi). The proportion of each repetitive fraction was calculated based on the number of reads assigned to each cluster relative to the total number of reads used in the final analysis, excluding putative contaminant sequences (mitochondrial and plastid DNA).

### Characterization of species satellitomes

Satellite DNA families were identified in nine *Alstroemeria* species using the TAREAN tool implemented in the RepeatExplorer2 server. This tool detects putative tandemly arranged repetitive sequences and reconstructs consensus sequences based on k-mer analysis, using the same dataset employed in the previous clustering analysis (Novák et al. 2017). The tandem organization of repeats was further validated by dot-plot analysis with default parameters in Geneious Prime v2021.1.1. The GC content of each satellite consensus monomer was also calculated in Geneious. Satellites were named according to the convention proposed by Ruiz-Ruano et al. (2016): the first three letters of the species, followed by “SAT”, the satellite number (based on abundance rank), and monomer length.

### Repeat-based and plastome phylogenies

The clustering analyses were performed based on repeat sequence similarity using the Alignment and Assembly-Free (AAF) method (Fan et al. 2015), applied to all reads identified as repeats or to tandem repeat reads identified by RepeatExplorer2. The AAF method infers phylogenies directly from unassembled genomic sequence data, bypassing the need for genome assembly and alignment. It computes the statistical properties of pairwise genomic distances, enabling parameter optimization and bootstrapping for robust phylogenetic inference. Here, we performed 100 bootstrap replicates to generate a consensus tree. The consensus was created with the ape package in R. Because repeat-abundance-based phylogenetic approaches (e.g., Dodsworth et al. 2015) rely on accurate genome-size measurements to obtain comparable absolute copy-number estimates across species, this strategy was not applicable here, as genome size data were not available for all analyzed taxa.

Additionally, to compare the topologies between the trees, plastomes were assembled for all the sampled species using the complete plastome of *Bomarea edulis* (KM233641.1) as a reference. The reads were mapped against this reference in Geneious v6.0.3 (Kearse et al. 2012) via the “Map to reference” function. The consensus plastomes were aligned with MAFFT (Katoh and Standley 2013). Maximum likelihood (ML) phylogenies were inferred with 1000 bootstrap replicates in Geneious v9.1.8 using the FastTree plugin (Price et al. 2009). The resulting trees were visualized and edited in FigTree (http://tree.bio.ed.ac.uk/software/figtree/).

### PCR amplification, probe labeling and FISH

For chromosomal mapping, we selected the most abundant satellite from *A. ligtu*, AliSAT1-285 (CL10), and the second most abundant from *A. hookeri*, AhoSAT2-361 (CL44). The primers were designed, sequences were amplified (see Supplementary Table 2), and probes were labeled by nick translation using DNA Polymerase I, DNase I (Thermo), and Cy3-dUTP (GE), following the protocol described by Ribeiro et al. (2021b). In addition, previously characterized satellites from *A. longistaminea*—AloSAT1, AloSAT5, AloSAT6A, and AloSAT7—were also mapped onto *A. urubiciensis*.

### *Slide preparation and fluorescence *in situ* hybridization (FISH)*

Root tips from *A. urubiciensis*, *A. hookeri*, and *A. ligtu* were pretreated with 0.2% colchicine at 10 °C for 24 h, fixed in ethanol–acetic acid (3:1, v/v) for 2 to 24 h at room temperature, and stored at −20 °C. In some cases, non-pretreated roots of *A. hookeri* and *A. ligtu* were also used. The mitotic preparations and CMA/DAPI staining were carried out as described by Vaio et al. (2018). Fixed root tips were washed in distilled water and digested at 37 °C for 60 min in a solution containing 2% (w/v) cellulase (Onozuka) and 20% (v/v) pectinase (Sigma).

FISH was performed following the protocol of Pedrosa et al. (2002). The hybridization mixture consisted of 50% (v/v) formamide, 10% (w/v) dextran sulfate, 2 × SSC, and 5–10 ng/μL of labeled probe. Slides were denatured at 75 °C for 5 min and hybridized overnight at 37 °C. For the heterologous hybridizations (i.e., *A. hookeri* satellite on *A. ligtu*, and vice versa), the protocol of Van-lume et al. (2019) was followed, using a hybridization buffer composed of 10% (w/v) dextran sulfate, 6× SSC, and 5–10 ng/μL of probe. In these cases, slides were also denatured at 75 °C for 5 min, with a final stringency of 40%. For all other satellite probes, the final stringency was ~76%.

The slides were examined and photographed using a Leica DMLB epifluorescence microscope. The images were captured with a Cohu CCD video camera using Leica QFISH software, and brightness and contrast were adjusted in Adobe Photoshop CS5 (v12.0). To improve chromosome pair identification, we followed the classification proposed by Ribeiro et al. (2021a), which groups chromosomes into five distinct categories based on size and morphology: large metacentric (M_L_), small metacentric (M_S_), large submetacentric (SM_L_), small submetacentric (SM_S_), and acrocentric (A) chromosomes of similar size.

## Results

### An overview of genomic composition in *Alstroemeria* based on individual analyses

The genome size estimate by flow cytometry for the species *A. monticola* was 2C = 53.11 picograms (1C = 25.97 Gbp) and for *A. urubiciensis* was 2C = 52.96 pg (1C = 25.90 Gbp). Based on the haploid karyotype length (HKL) of *A. violacea* (118.25 µm; Baeza and Toro 2021), we estimated its approximate genome size by comparison with data from *A. longistaminea* (Ribeiro et al. 2021a; Nascimento et al. 2025a), resulting in an estimated genome size of approximately 1 C = ~28 Gbp for *A. violacea*.

In the individual repeatome analyses, all species exhibited similar overall repeat compositions, with repetitive elements accounting for between 61.73% of the genome in *A. monticola* and 68.79% in *A. exerens*. The most abundant repetitive element across all species was LTR-Tekay, a member of the Ty3/gypsy superfamily from the Chromovirus lineage, comprising up to 39.91% of the genome in *A. philippii* and 30.63% in *A. pulchra*. Ty3/gypsy Retand, a non-Chromovirus lineage, showed particularly high abundance in *A. pulchra* (11.03%), nearly twice that observed in the other species. Within the Ty1/copia superfamily, the most prominent lineage was Tork, accounting for between 2.86 and 4.30% of the genome (Table 2 and Supplementary Fig. 1).

The Chilean species generally displayed a higher abundance of satellite DNA than the Brazilian species, with *A. ligtu* showing the highest proportion (3.42%) and *A. violacea* the lowest (0.56%). Among the Brazilian group, satellite content ranged from 0.23% in *A. psittacina* to 1.70% in *A. urubiciensis* (Table 2). Similarly, rDNA was more abundant in the Chilean species, exemplified by *A. pulchra* (1.17%) compared to *A. psittacina* (0.18%) (Table 2). All data from the individual analyses are summarized in Table 2 and Supplementary Fig. 1. Due to the lack of available specimens and published data, karyotypic and genome size estimates could not be obtained for *A. exerens*.

### Comparative clustering analysis revealed similar genomic landscapes among *Alstroemeria* species

**Fig. 1:**
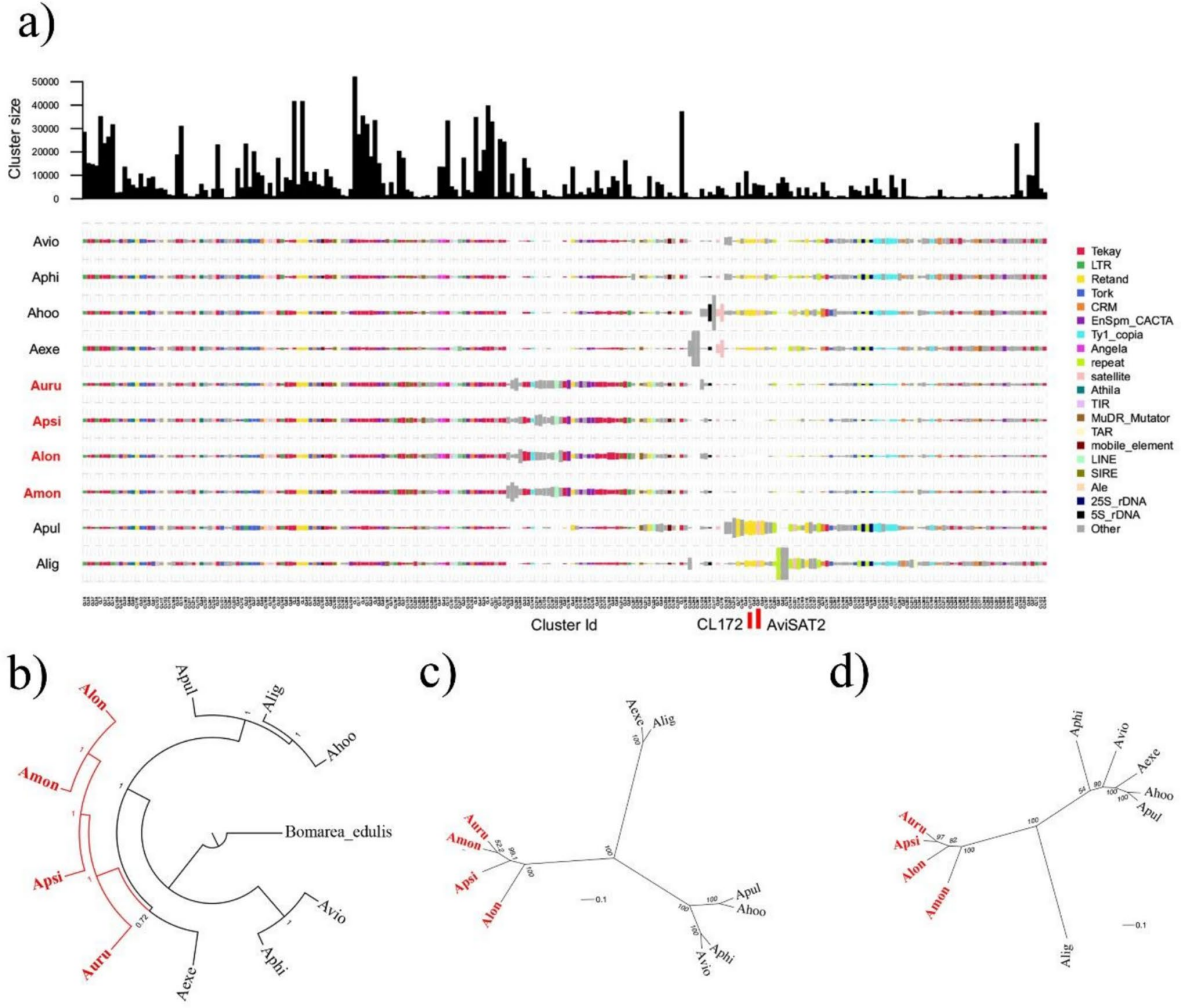
Overview of the comparative analysis of repetitive elements and phylogenetic inferences across ten species of the genus *Alstroemeria*. **a** RepeatExplorer comparative analysis with each species represented by their acronyms: Avio (*A. violacea*), Aphi (*A. philippii*), Ahoo (*A. hookeri*), Aexe (*A. exerens*), Auru (*A. urubiciensis*), Apsi (*A. psittacina*), Alon (*A. longistaminea*), Amon (*A. monticola*), Apul (*A. pulchra*), and Alig (*A. ligtu*). The X-axis shows the different repeat clusters, indicated by distinct colors. **b** Phylogenetic tree inferred from the alignment of plastome sequences assembled by reference. The tree is rooted in *Bomarea edulis*, a sister species of *Alstroemeria* and the only species of the genus described for Brazil. **c** AAF tree derived from the similarity of all repetitive sequences, showing a clear separation between the Brazilian and Chilean groups, consistent with the known geographic structure of the species. **d** AAF tree generated using exclusively tandem repeats, exhibiting a distinct topology suggesting faster evolution of satellite DNAs. In **c** and **d** branch length represents k-mer distances and numbers at internal nodes indicate bootstrap support values. The Brazilian species (*A. urubiciensis, A. longistaminea, A. monticola,* and *A. psittacina*, collected in Argentina) are shown in red; the remaining six species are Chilean

**Fig. 2:**
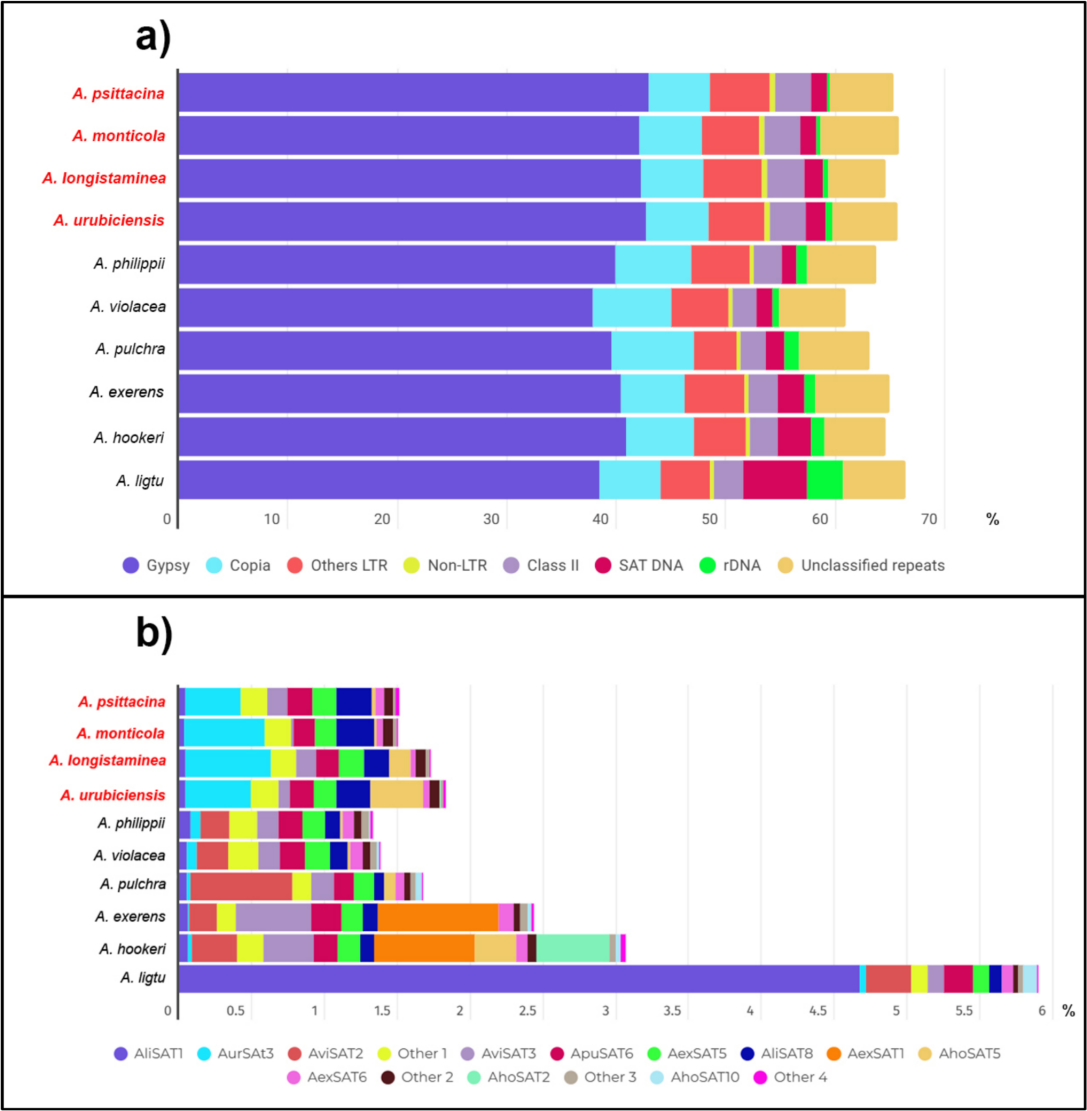
Genomic proportions of repetitive sequences in *Alstroemeria* species from the RE comparative analysis. Brazilian species are highlighted in *red*. **a** Percentage of the genome composed of repetitive sequences (X-axis), with distinct types shown in distinct colors. **b** Satellite composition of the species with the total proportion of satellites represented by the X-axis

The comparative analysis of ten *Alstroemeria* species—Chilean and Brazilian/Argentinean—yielded 286 repeat clusters and confirmed the general trends observed in the individual analyses. All major repeat types were shared among species and LTR-Tekay (Ty3/gypsy) remained the most abundant element (Supplementary Table 1; Figs. 1a, 2a). All clusters of this element were present in every species, though some in small quantities. Ty3/gypsy Retand was the second most abundant, followed by CRM. Again, all clusters of these elements were shared across species. In the Ty1/copia group, Tork was the most abundant (Supplementary Table 1; Figs. 1a, 2a). Other non-LTR retroelements, such as Pararetrovirus and LINEs, were also detected in all species but contributed to less than 1% of the genome. DNA transposons were also shared among all species with relatively uniform representation (Supplementary Table 1; Fig. 1a).

Some variation in cluster distribution was observed. For example, Retand clusters CL48, CL82, CL172 were exclusive to Chilean species and absent in all four Brazilian species analyzed. Similarly, certain satellite DNA clusters were group-specific: AviSAT2 (CL91) was found only in the Chilean species, and AhoSAT10 (CL226) was virtually absent in the Brazilian species, except for *A. longistaminea*, in which it was present at a very low proportion (0.0003209%). The repeat composition patterns were more homogeneous among the four Brazilian/Argentinean species. Some Chilean species showed greater variation, although species such as *A. philippii* and *A. violacea*, and *A. exerens* and *A. hookeri*, exhibited more similar profiles (Figs. 1a, 2a).

### Repetitive sequence similarities reflect phylogenetic relationships in *Alstroemeria*

The plastid phylogeny supported a Brazilian radiation nested within the Chilean lineage, and placed, with low support, *A. exerens* as sister to the Brazilian clade (Fig. 1b), in agreement with previous phylogenetic studies (Chacón et al. 2012). Phylogenomic analyses based on repetitive DNA consistently recovered the Brazilian and Chilean groups regardless of the method employed (Fig. 1c, d), indicating that, at broader evolutionary scales, patterns of repeat divergence broadly parallel species divergence.

Both phylogenomic approaches based on repetitive DNA—AAF analyses using total or tandem repeats—consistently recovered the Brazilian lineage (Fig. 1c, d). However, the internal branching varied among methods and when compared to the plastid topology, suggesting that the limited repetitive divergence among these species was not sufficient to support phylogenetic inference within clades or a lack of phylogenetic signal at this level. Nevertheless, plastome and repeat-based phylogenies were congruent in recovering the separation between the Chilean and Brazilian groups and in indicating lower divergence among the Brazilian species compared to the more diverse Chilean grade.

Within lineages, patterns of satellite DNA composition showed partial correspondence with plastid relationships. For example, *A. violacea* and *A. philippii* share similar satellite profiles (Fig. 2b and 1d) and are distinct from the remaining Chilean species, consistent with their close association within the Chilean clade in the plastid phylogeny (Fig. 1b). In contrast, *A. exerens* represents a notable case of discordance between plastid and repeat-based analyses. Although it is less related to *A. ligtu* in the plastid tree (Fig. 1b), both species cluster together in repeat-based analyses (Fig. 1c). Importantly, this clustering is not reflected in their satellite compositions, which differ markedly between the two species (Figs. 1d, 2b).

### Conservation of satellite repeats between Brazilian and Chilean species of *Alstroemeria*

SatDNA characterization using TAREAN employed the same dataset as the individual analysis for nine species. Additionally, a database containing previously identified satellites from *A. longistaminea* was included. A total of 82 clusters of tandem repeats were identified across all species (Table 3). Specifically, 17 clusters were found in *A. pulchra*, 13 in *A. exerens*, 12 in *A. hookeri*, 10 in *A. urubiciensis*, nine in *A. ligtu*, six in both *A. philippii* and *A. violacea*, five in *A. monticola*, and four in *A. psittacina*. Monomer sizes ranged from 22 bp (AhoSAT1-22) to 4757 bp (AliSAT7-4757), with AT content varying from 25.6% (*A. pulchra*—ApuSAT16-39) to 69.8% (*A. psittacina*—ApsSAT4-2545) (Table 3). The genomic abundance of these satellites also varied from 0.01% (e.g., AexSAT12-331, AhoSAT12-142, ApsSAT4-2545) to 1.98% (AliSAT1-285).

**Table 3:** Satellite DNAs identified for all of species after individual analysis using the TAREAN tool from RepeatExplorer

Satellites	Genomic abundance (%)	Monomer size (pb)	A + T content (%)	Confidence	Similarity with satellites DNA of *A. longistaminea* (%)
*A. exerens*					
AexSAT1-33	0.72	33	42.4	High	
AexSAT2-167	0.49	167	58.1	Low	
AexSAT3-3619	0.17	3619	57.7	Low	
AexSAT4-191	0.11	191	50.3	Low	
AexSAT5-202	0.11	202	52	Low	
AexSAT6-201	0.09	201	47.8	Low	
AexSAT7-2335	0.09	2335	60.9	Low	
AexSAT-8–2793	0.07	2793	60.1	High	
AexSAT9-154	0.06	154	49.4	Low	74.60% (AloSAT6A)
AexSAT10-2089	0.04	2089	58.8	Low	
AexSAT11-3979	0.03	3979	60.1	High	
AexSAT12-331	0.01	331	33.5	Low	
AexSAT13-37	0.01	37	45.9	Low	
*A. hookeri* subs. *hookeri*					
AhoSAT1-22	0.71	22	46	High	
AhoSAT2-361	0.51	361	52.4	High	
AhoSAT3-2467	0.35	2467	52.4	Low	67.18% (AloSAT7)
AhoSAT4-1513	0.31	1513	63.3	High	
AhoSAT5-103	0.28	103	31.1	Low	
AhoSAT6-201	0.15	201	49.3	Low	
AhoSAT7-348	0.11	348	51.7	Low	
AhoSAT8-104	0.09	104	53.8	Low	
AhoSAT9-2337	0.07	2337	60.8	Low	
AhoSAT10-154	0.04	154	50	Low	81.43% (AloSAT6A)
AhoSAT11-192	0.02	192	42.7	Low	
AhoSAT12-142	0.01	142	51.4	Low	
*A. urubiciensis*					
AurSAT1-869	0.43	869	67.4	Low	43.57% (AloSAT5)
AurSAT2-4573	0.38	4573	53.9	Low	
AurSAT3-109	0.26	109	52.3	Low	
AurSAT4-312	0.17	312	55.8	Low	
AurSAT5-3591	0.13	3591	58.5	Low	
AurSAT6-155	0.1	155	51	Low	67.82% (AloSAT6A)
AurSAT7-1678	0.1	1678	67.1	Low	94.73% (AloSAT7)
AurSAT8-331	0.05	331	49.2	Low	
AurSAT9-167	0.01	167	58.7	Low	
AurSAT10-36	0.01	36	66.7	Low	
*A. ligtu* subs. *ligtu*					
AliSAT1-285	1.98	285	33	High	
AliSAT2-5970	0.58	5970	60.4	Low	
Ali SAT3-392	0.24	392	49	Low	
Ali SAT4-98	0.2	98	46.9	Low	
Ali SAT5-3655	0.16	3655	58.1	Low	
Ali SAT6-927	0.11	927	66.8	High	68.26% (AloSAT7)
Ali SAT7-4757	0.07	4757	66.1	High	
Ali SAT8-2713	0.06	2713	60.6	Low	
Ali SAT9-192	0.02	192	43.2	Low	
*A. monticola*					
AmoSAT1-2639	0.25	2639	49.8	Low	
AmoSAT2-1488	0.22	1488	60.1	Low	
AmoSAT3-3623	0.12	3623	58.7	Low	
AmoSAT4-152	0.08	152	50.7	Low	79.20% (AloSAT6B)
AmoSAT5-142	0.02	142	52.8	Low	78.60% (AloSAT6B)
*A. philippii* subs. *philippii*					
AphSAT1-200	0.18	200	48.5	Low	
AphSAT2-102	0.16	102	28.4	Low	
AphSAT3-1925	0.15	1925	67	High	67.18% (AloSAT7)
AphSAT4-159	0.07	159	57.2	Low	80.87% (ALoSAT6A)
AphSAT5-1978	0.05	1978	58.4	Low	
AphSAT6-192	0.02	192	43.7	Low	
*A. psittacina*					
ApsSAT1-1678	0.15	1678	67.2	High	49.1% (AloSAT7)
ApsSAT2-861	0.04	861	40.0	Low	
ApsSAT3-1039	0.03	1039	66.5	Low	
ApsSAT4-2545	0.01	2545	69.8	Low	
*A. pulchra* subs. *pulchra*					
ApuSAT1-159	0.31	159	56.6	Low	74.10% (AloSAT6A)
ApuSAT2-145	0,31	145	31	Low	
ApuSAT3-101	0.31	101	30.7	Low	
ApuSAT4-154	0.23	154	52.6	Low	79.64% (AloSAT6A)
ApuSAT5-4156	0.20	4156	47.1	Low	
ApuSAT6-3593	0.15	3593	58.1	Low	
ApuSAT7-180	0.13	180	38.9	Low	
ApuSAT8-104	0.13	104	53.8	Low	
ApuSAT9-199	0.13	199	47.2	Low	
ApuSAT10-416	0.11	416	51.4	High	
ApuSAT11-58	0.10	58	34.5	Low	
ApuSAT12-1497	0.08	1497	62.9	Low	
ApuSAT13-304	0.08	304	35.2	Low	
ApuSAT14-425	0.07	425	34.6	Low	
ApuSAT15-379	0.05	379	62.8	High	
ApuSAT16-39	0.03	39	25.6	Low	
ApuSAT17-98	0.01	98	33.7	Low	
*A. violacea*				Low	
AviSAT1-200	0.19	200	49.0	Low	
AviSAT2-137	0.15	137	30.1	Low	
AviSAT3-1971	0.13	1971	67.1	High	69.53% (AloSAT7)
AviSAT4-304	0.04	304	36.2	Low	
AviSAT5-98	0.03	98	35.7	Low	
AviSAT6-192	0.02	192	42.7	Low	

The confidence parameter, automatically generated by TAREAN, estimates the probability of a cluster being a genuine satellite (high confidence) or a complex/ambiguous repeat (low confidence). Percentage of similarity generated by the TAREAN tool of RepeatExplorer

Alignments of the satellite sequences revealed that all species had at least one cluster showing similarity to at least one of three satDNA families from *A. longistaminea*. For instance, AexSAT9-154 (74.60% similarity), AhoSAT10-154 (81.43%), AurSAT6-155 (67.82%), AphSAT4-159 (80.87%), ApuSAT1-159 (74.10%), and ApuSAT4-154 (79.64%) showed similarity to AloSAT6A. Satellites AmoSAT4-152 (79.20%) and AmoSAT5-142 (78.60%) were similar to AloSAT6B, while AurSAT7-1678 (94.73%), AliSAT6-927 (68.26%), AphSAT3-1925 (67.18%), and AviSAT3-1971 (69.53%) showed similarity to AloSAT7 (Table 3).

Annotation of tandem repeats from the comparative analysis revealed 16 satDNA families, most of which are shared among all analyzed species (Fig. 2b; Supplementary Table 3). The Brazilian species displayed highly similar abundances of all 16 families. In contrast, four satDNA families (AviSAT2-137, AexSAT1-33, AhoSAT2-361, and AhoSAT10-154) were highly abundant in the Chilean species but were either absent or present in very low amounts in the Brazilian species. While the Chilean species shared most satDNA families, they exhibited considerable variation in satellite composition. For example, AhoSAT2, the second most abundant in *A. hookeri*, was not detected in the other four Chilean species. Overall, *A. philippii* and *A. violacea* shared a similar satellitome composition, as observed among Brazilian species, a pattern supported by the AAF tree based on tandem repeat sequences (Figs. 1c, d, 2b; Supplementary Table 3).

Some satellites identified by TAREAN were annotated by RepeatExplorer as being associated with transposable elements. These include AhoSAT5-103, AhoSAT8-104, AphSAT2-102, ApuSAT2-145, and AviSAT2-137, which are related to the Retand lineage; AurSAT3-109, AurSAT8-331, and AliSAT8-2713, related to Tekay; AliSAT2-5970 and ApuSAT12-1497, related to Angela; AliSAT5-3655, related to CRM; AmoSAT1-2639, ApuSAT13-304, and AviSAT4-304, related to Harbinger; AmoSAT2-1488, related to CACTA; and ApsSAT2-861, related to Reina.

### Chromosome and sequence conservation of *A. longistaminea* repeats in *A. urubiciensis*

Four satellites previously described and mapped in *A. longistaminea* (Ribeiro et al. 2021b) were hybridized onto the karyotype of *A. urubiciensis* to investigate their conservation in chromosomal distribution. Satellites AloSAT6A and AloSAT7 were selected due to their 67–94% sequence similarity with satellites of *A. urubiciensis*, and conservation to other *Alstroemeria* species as revealed by TAREAN analysis. Furthermore, AloSAT1 and AloSAT5 were chosen because they are abundant and showed a heterochromatic distribution in *A. longistaminea*, similarly to AloSAT7. AloSAT6A, on the other hand, showed an euchromatic distribution in *A. longistaminea* (Ribeiro et al. 2021b).

**Fig. 3:**
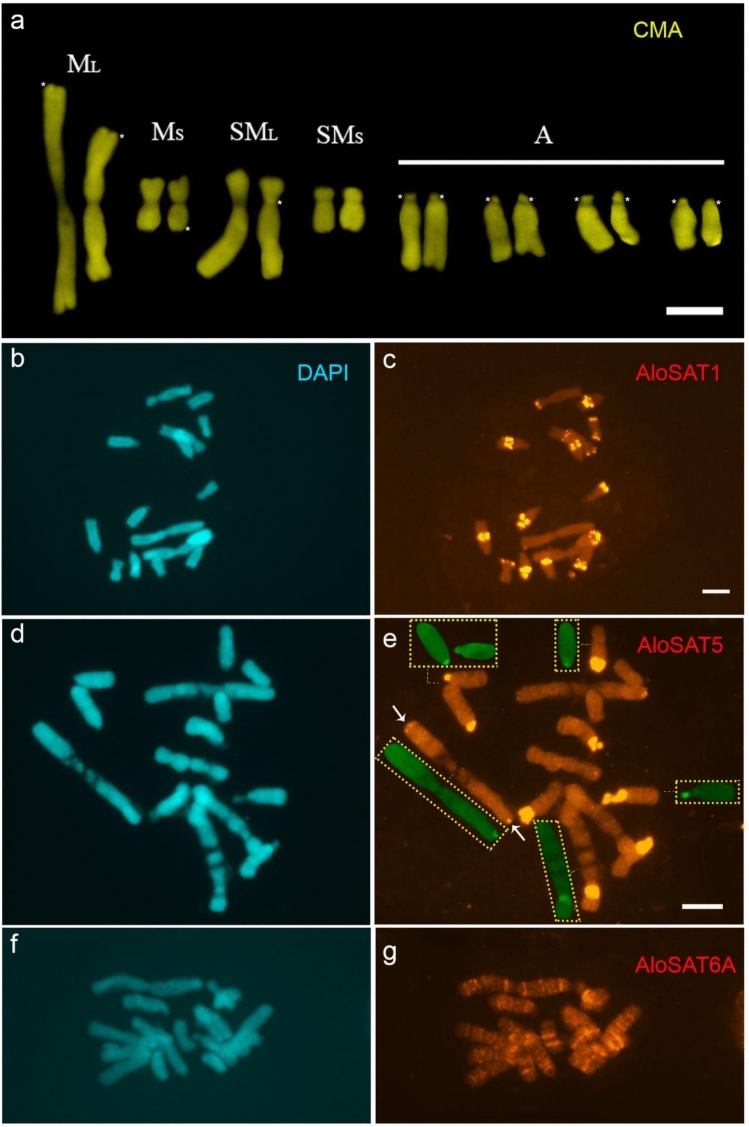
CMA/DAPI banding pattern (**a**, **b**) and different satDNAs of *Alstroemeria longistaminea* (**b**–**g**) hybridized to *A. urubiciensis* chromosomes. In **d**–**g**, the chromosomes were photographed with a 100× objective, while in **a**–**c**, with a 63× objective. In a, a karyogram is shown to better highlight the CMA⁺ bands indicated by an *asterisk*. In **e**, the insets show some 35S rDNA sites (in *green*) that co-localize with the AloSAT5 sites. Scale bar = 10 µm

Hybridization with AloSAT1 produced clear signals on all chromosomes except for the M_L_ pair. This satellite labeled the interstitial regions of all acrocentric chromosomes, the terminal regions of three of the four arms of the M_S_ chromosomes, the subtelomeric region of the SM_S_ pair, and the terminal region of the short arm of the SM_L_ pair, all appearing as dot-like signals (Fig. 3b, c). AloSAT5 hybridized as blocks on the short arms of seven out of eight acrocentrics, showed dot-like signals on both arms of the M_L_ pair, and produced a heteromorphic centromeric signal on the SM_L_ pair (Fig. 3d, e). The AloSAT5 satellite co-localized with seven of the eight 35S rDNA sites on the short arms of the acrocentrics, with the site at the end of the M_L_ pair, and with one of the sites on the SM_L_ pair (Fig. 3—insets in e showing 35S rDNA sites).

The satellite AloSAT6A displayed G-banding-like labeling along all chromosomes, particularly prominent in the M_L_ pair (Fig. 3f, g), conserving its euchromatic distribution. In contrast, AloSAT7 did not produce detectable hybridization signals on *A. urubiciensis* chromosomes (data not shown). Although AloSAT7 shows high similarity (94.73%) with AurSAT7-1678 from *A. urubiciensis*, it has a complex structure in *A. longistaminea*, where it was centromeric in most chromosomes but also included degenerated, interspersed telomeric repeats (Ribeiro et al. 2021b). Its low genomic abundance in *A. urubiciensis* (0.1%) should be sufficient for detection, since AloSAT6A showed similar abundance and was detected in a disperse distribution, and AloSAT1 and AloSAT5, which generated clear signals, were not even detected among the most abundant, annotated repeats of *A. urubiciensis* (Table 3). It is, thus, possible that the AloSAT7 probe used was not adequate to generate a detectable signal by FISH due to its complex sequence structure.

### Satellites of Chilean *A. hookeri* and *A. ligtu* are distributed in major blocks across the karyotype

**Fig. 4:**
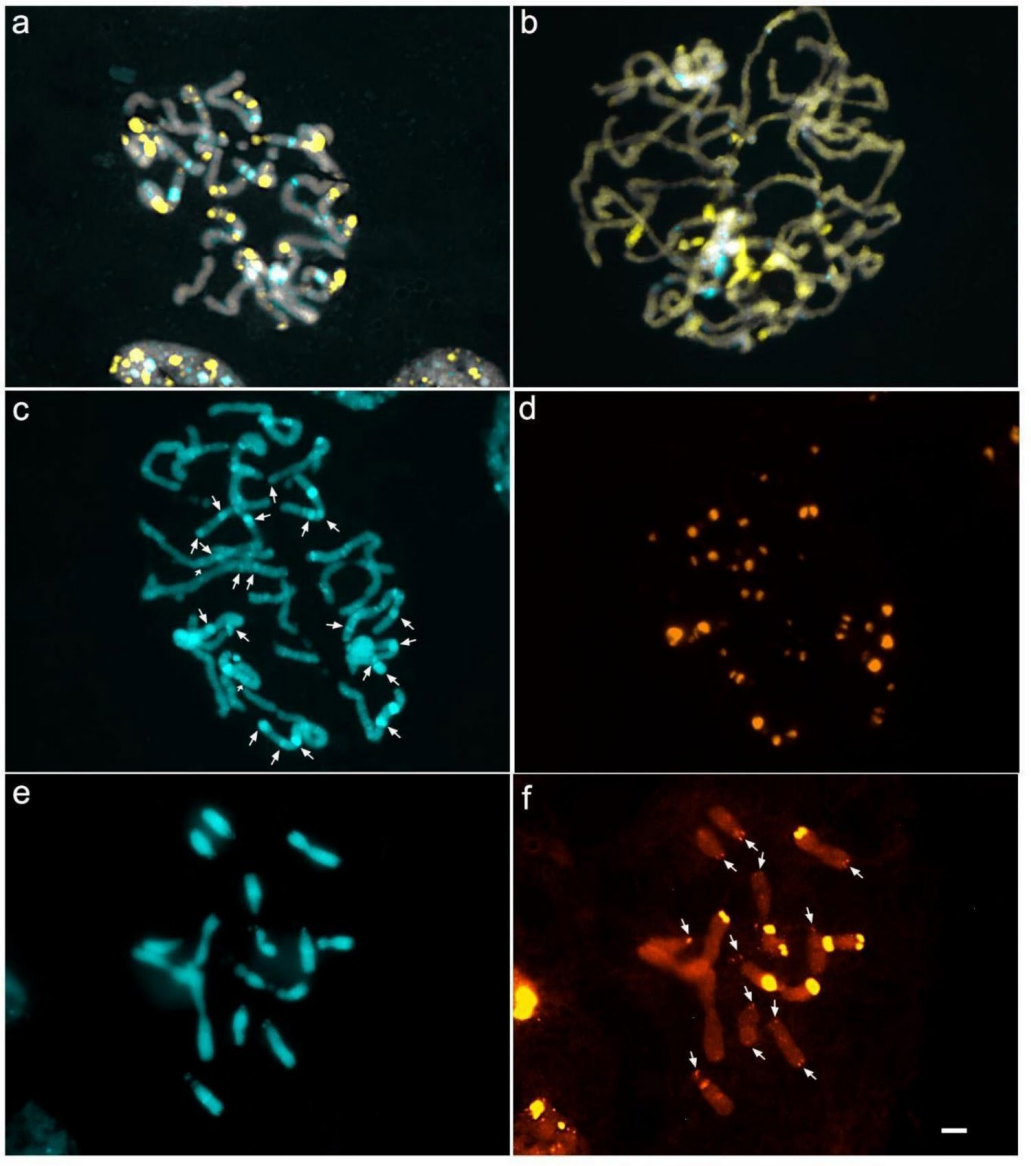
CMA/DAPI banding and fluorescent *in situ* hybridization (FISH) on non-pretreated mitotic chromosomes of Chilean *Alstroemeria* species. **a**, **b** CMA/DAPI banding, showing large GC-rich heterochromatic blocks in *yellow* and some AT-rich in *blue* in *A. ligtu* (**a**) and *A. hookeri* (**b**). **c**–**d** FISH mapping of the AliSAT1-285 satellite sequence from *A. ligtu* on *A. ligtu* chromosomes. White arrows in (**c**) show DAPI-stained heterochromatic blocks that co-localize with AliSAT1 signals. **e**–**f** FISH mapping of the AhoSAT2-361 satellite sequence from *A. hookeri* on *A. hookeri* chromosomes. *White arrows* in **f** indicate small hybridization signals of the satellite. Scale bar = 10 µm

We selected satellite AhoSAT2-361 from *A. hookeri* and AliSAT1-285 from *A. ligtu* to investigate their potential association with prominent heterochromatic bands revealed by CMA/DAPI staining (Fig. 4a, b). AhoSAT2-361, which is also present in other Chilean species (except for *A. ligtu* and *A. violacea*) (Fig. 2b), is classified as satellite DNA (whereas AhoSAT1-22 is a minisatellite). It has an AT content of 52.4% and accounts for 0.51% of the *A. hookeri* genome (Table 3 Supplementary Material). AliSAT1-285 is the most abundant tandem repeat in *A. ligtu* (1.98%), is GC-rich (AT content of 33%) and is also well represented in the genomes of other *Alstroemeria* species (Fig. 2b; Table 3).

AliSAT1 revealed large interstitial and terminal blocks in *A. ligtu* (Fig. 4c, d), mostly colocalized with heterochromatic bands, but not with the most prominent DAPI^+^ bands (Fig. 4a, c, d). When hybridized to *A. hookeri*, AliSAT1 produced faint signals at some chromosome termini (Supplementary Material Fig. 3d). AhoSAT2 generated only terminal signals in *A. hookeri*, with three chromosome pairs showing stronger labeling (Fig. 4e, f). In *A. ligtu*, AhoSAT2 hybridization produced terminal signals on nearly all chromosomes, along with a few interstitial signals (Supplementary Material Fig. 3b).

## Discussion

This study provides the first in silico characterization of the repetitive fraction in six Chilean and three Brazilian/Argentinean *Alstroemeria* species, offering the first comparative overview between these lineages. The comparative analysis revealed a pattern of low sequence turnover, with the majority of repeat clusters—including all major retrotransposon lineages—being shared across all species. The few exceptions to this widespread conservation were informative: certain Retand clusters (e.g., CLs 48, 82, 172) and satellite DNAs (e.g., AviSAT2, AhoSAT2) showed lineage-specific distributions or abundances. Notably, the satellite AviSAT2 (CL91) was detected exclusively in the Chilean species, suggesting its loss in the Brazilian lineage.

Within the Chilean group, our plastome phylogeny, although including a limited number of species, suggests a close relationship between *A. philippi* and *A. violacea*. This phylogenetic relationship is corroborated by their highly similar repeatome profiles and by AAF analyses, which cluster them together based on both overall and tandem repeat sequences. This relationship is further supported by Baeza and Toro-Núñez (2021) and Finot et al. (2018a, b), who distinguished *A. violacea* and *A. philippii* primarily by the intensity of the purple hue in their tepals and their geographic distributions. Our phylogenetic results, together with data on repetitive sequence composition, indicate that repetitive DNA analyses can either mirror or deviate from plastid-based relationships, depending on the lineage and on the genomic components considered. While some species show strong congruence between plastome phylogeny and repeatome structure, others reveal marked discordance, highlighting the heterogeneous dynamics of repetitive DNA evolution, namely TEs vs. satDNAs, within the genus.

To evaluate whether satellite DNA conservation in *Alstroemeria* also extends to chromosomal distribution, we mapped abundant satellites from *A. longistaminea* onto the *A. urubiciensis* karyotype, which showed CMA^+^ bands restricted to the short arms of acrocentric pairs (Nascimento et al. 2025b). AloSAT1 and AloSAT6A showed similar patterns in *A. urubiciensis*. The overall similarity between these species—also reported for two other Brazilian *Alstroemeria* (Ribeiro et al. 2021b)—suggests that conservation among Brazilian species encompasses not only karyotype structure but also satDNA abundance and chromosomal distribution. Some variation occurs, however, in *A. urubiciensis*, AloSAT5 co-localizes with 35S rDNA on five chromosome pairs, whereas in *A. longistaminea* it was restricted to one terminal site and B chromosomes, also intercalated with 35S rDNA repeats. This pattern suggests that the association between AloSAT5 and rDNA potentially explains its lower conservation due to the known dynamism of rDNA regions (Ribeiro et al. 2021a).

The Chilean species generally exhibit higher proportions of satellite DNA than the analyzed the Brazilian species, although the overall diversity of satDNA families, based on the number of distinct families appears to be equivalent in both groups. Notably, one of the species with the highest proportion of C-bands, *A. ligtu* (approximately 12.9% of the chromosomal complement; Buitendijk and Ramanna 1996), also show higher satellite DNA content (3.42%), with their major satellite (AliSAT1-285) colocalizing with large heterochromatic blocks. Conversely, *A. philippii*, a species with one of the lowest C-band contents (ca. 2.0%), exhibited a lower proportion of satellite DNA (0.63%). This association, together with the marked longitudinal heterochromatin differentiation described for Chilean taxa (Buitendijk and Ramanna 1996), suggests that satellite DNA abundance contributes significantly to karyotypic divergence between the Chilean and Brazilian lineages. Additionally, the distribution patterns of satellite DNAs also appear to play an important role in this longitudinal chromosomal differentiation. In the Chilean species *A. ligtu* and *A. hookeri*, satellites form large interstitial and terminal blocks that broadly coincide with heterochromatic regions, despite differences in satellite identity and abundance. In contrast, in *A. longistaminea*, seven out of ten satellites were primarily dispersed (Ribeiro et al. 2021b). A similar dispersed pattern was also reported in *Bomarea edulis*, a large genome species from a sister genus, which has few heterochromatic bands (Nascimento et al. 2025a). Taken together, these observations indicate that, in Chilean *Alstroemeria*, satellite DNA diversification tends to occur within conserved heterochromatic domains, whereas the Brazilian species exhibit a more dispersed organization of satellite repeats. While our data do not allow direct inference of the mechanisms underlying these patterns, they are consistent with the view that chromosomal architecture influences the spatial dynamics of satellite DNA evolution, with replacement and homogenization of repeats within heterochromatic domains or dispersal of smaller tandem repeat blocks in large genomes.

Our results indicate that, despite an evolutionary divergence of approximately ~9,2 million years between Chilean and Brazilian *Alstroemeria*, the analyzed species exhibit a remarkably conserved genomic architecture, with only a few lineage-specific amplifications of certain repetitive elements. The variations were generally minimal, except for certain satellite DNAs and a notable fourfold increase of the Ty3/Retand lineage in *A. pulchra*. This conservation contrasts with the faster repeat turnover typical of small-genome herbaceous lineages, such as *Macroptilium* (Montenegro et al. 2025), and resembles patterns observed in long-lived or woody taxa. For example, *Cenostigma* shows stable TE composition and chromosomal organization, with only subtle satellite changes (Castro et al. 2023), and *Populus* exhibits conserved repeat fractions aside from a few satellites and one Ty3/gypsy lineage (Usai et al. 2017). These comparisons support the hypothesis that perennial, long-lived species tend to experience slower repeat renewal and genomic change than short-lived herbs (Leitch and Leitch 2013; Novák et al. 2020a).

Interestingly, although *Alstroemeria* is an herbaceous lineage, its repeat dynamics resemble those of long-lived perennial trees, showing low turnover and slow genome evolution—possibly linked to its large genome size. According to Novák et al. (2020a), once genomes exceed ~10 Gb, repeat content stabilizes and is dominated by a heterogeneous and ancient set of elements, with minimal diversification of new families. *Alstroemeria* follows this pattern (1C ~ 20–28 Gb; ~61–68% repeats), with lineages such as Tekay prevailing and minimal incorporation of new families. This reflects low deletion rates and constrained evolutionary dynamics, consistent with the genome obesity hypothesis (Bennetzen and Wang 2014; Kelly et al. 2015), which proposes that large genomes retain repetitive DNA due to slow turnover rates. The limited diversification of repeat families among *Alstroemeria* species further reinforces this slow-renewal pattern typical of giant genomes, highlighting how genome size and turnover rates shape evolutionary trajectories over millions of years.

Comparisons among *Alstroemeria*, *Allium* and *Fritillaria* reveal, however, that the dynamics of repetitive elements in large genomes do not follow a single evolutionary pattern. In *Alstroemeria*, despite ~9.2 Myr of divergence between the Chilean and Brazilian lineages, the repeatome remains highly conserved, except for the abundance of some heterochromatic satDNAs. *Allium*, although also possessing massive genomes (*A. cepa*, 1 C = 16.75 pg; *A. sativum*, 1 C = 16.25 pg; e *A. ursinum*, 1 C = 31.45 pg; Peška et al. 2019), shows a more dynamic repeat landscape in its ~41 Myr diversification (Xie et al. 2020), with species differing substantially in their repeat composition. *Fritillaria*, with some of the largest plant genomes (~ 85 Gb), also show higher diversity of repeats among species, suggesting independent gradual accumulation of old, heterogeneous repeats (Kelly et al. 2015).

Together, these patterns show that the evolution of large genomes depends on the interaction between evolutionary time, life-history traits, and the balance between amplification and deletion. While *Allium* exhibits slow yet ongoing renewal, *Fritillaria* accumulates ancient sequences with little turnover, and *Alstroemeria* maintains remarkable repeatome stability over millions of years, despite heterochromatin differentiation. In this context, the extremely low rate of repeat renewal and limited diversification contribute to the maintenance of a structurally stable genome in the species analyzed here and may impact the evolution of the group, with a higher probability of genome compatibility after hybridization (Castro et al. 2023).

## Conclusion

We present the first in-depth comparative analysis of the repetitive fraction in *Alstroemeria* species from the Chilean and Brazilian groups. Our results show that, despite the evident longitudinal karyotypic differentiation between these groups, they exhibit a remarkably conserved overall genome composition, with only minor variation—primarily in the abundance of a few tandem repetitive elements. These findings may be primarily related to its giant genome sizes and suggest that the differences in heterochromatin distribution observed between the Chilean and Brazilian species are not due to the presence of exclusive satellite families, but rather to the differential amplification and chromosomal organization of shared satellite DNAs. In particular, the formation of large heterochromatic blocks in the Chilean species, in contrast to the more dispersed patterns observed in the Brazilian species, appears to play a central role in their karyotypic divergence. Finally, these findings provide a broader perspective on genome evolution in the context of low turnover, revealing how long-term genomic stability can persist even in highly diverse and geographically isolated herbaceous lineages.

## Supplementary Information

Below is the link to the electronic supplementary material.

Supplementary file1 (DOCX 380 KB)

## Data Availability

Raw sequence data resulting from low-coverage sequencing for the species analyzed here is available in the National Center for Biotechnology Information (NCBI) repository. Accession numbers are available in Table 1.

## References

[CR2] Assis MC (2002) Novas espécies de *Alstroemeria* L. (Alstroemeriaceae) de Minas Gerais, Brasil. Rev Bras Bot 25:177–182. 10.1590/S0100-8404200200020000

[CR3] Baeza C, Toro-Núñez O (2021) El cariotipo fundamental de *Alstroemeria violacea* Phil. (Alstroemeriaceae). Gayana Bot 78:196–199. 10.4067/S0717-66432021000200196

[CR7] Baeza CM, Ruiz E, Negritto M (2010) Comparative karyotypic analysis in the *Alstroemeria hookeri* Lodd. (Alstroemeriaceae) complex sensu Bayer (1987). Genet Mol Biol 33(1):119–124. 10.1590/S1415-47572010005000012 21637614 10.1590/S1415-47572010005000012 PMC3036094

[CR8] Baeza CM, Ruiz E, Rosas M (2012) Es *Leontochir ovallei* Phil. (Alstroemeriaceae) um gênero distinto a *Bomarea* Mirbel? Consideraciones citológicas. Agro- Ciencia Chilean J Agric Aninn Sci 28(1):63–67

[CR10] Baeza CM, Finot V, Ruiz E, Carrasco P, Novoa P, Stuessy T, González A (2016) Comparative karyotypic analysis and cytotaxonomy in the *Alstroemeria ligtu* L. (Alstroemeriaceae) complex of Chile. Braz J Bot 39:305–313. 10.1007/s40415-015-0220-4

[CR12] Bennetzen JL, Wang H (2014) The contributions of transposable elements to the structure, function, and evolution of plant genomes. Annu Rev Plant Biol 2014:505–530. 10.1146/annurev-arplant-050213-035811 10.1146/annurev-arplant-050213-035811 24579996

[CR13] Bourque G, Burns KH, Gehring M, Gorbunova V, Seluanov A, Hammell M, Imbeault M, Izsvák Z, Levin HL, Macfarlan TS et al (2018) Ten things you should know about transposable elements. Genome Biol 19:199–210. 10.1186/s13059-018-1577-z 30454069 10.1186/s13059-018-1577-z PMC6240941

[CR14] Buitendijk JH, Ramanna MS (1996) Giemsa C-banded karyotypes of eight species of *Alstroemeria* L. and some of their hybrids. Ann Bot 78:449–457. 10.1006/anbo.1996.0141

[CR15] Buitendijk JH, Boon EJ, Ramanna MS (1997) Nuclear DNA content in twelve species of *Alstroemeria* L. and some of their hybrids. Ann Bot 79:343–353. 10.1006/anbo.1996.0345

[CR16] Castro N, Mata-Sucre Y, Carvalho-Sobrinho J, Marques A, Queiroz RT, Souza G (2023) Genomic stability in *Cenostigma* Tul., (Caesalpinioideae, Fabaceae): causes and consequences. Bot J Linn 204(2):137–151. 10.1093/botlinnean/boad043

[CR17] Chacón J, Assis MC, Meerow AW, Renner SS (2012) From East Gondwana to Central America: historical biogeography of the Alstroemeriaceae. J Biogeogr 39:1806–1818. 10.1111/j.1365-2699.2012.02749.x

[CR19] Dhiman MR, Kashyap B (2022) *Alstroemeria*: conservation, characterization, and evaluation. In: Datta SK, Gupta YC (eds) Floriculture and ornamental plants. handbooks of crop diversity: conservation and use of plant genetic resources. Springer, Singapore. 10.1007/978-981-15-3518-5_7

[CR20] Dodsworth S, Chase MW, Kelly LJ et al (2015) Genomic repeat abundances contain phylogenetic signal. Syst Biol 64:112–126. 10.1093/sysbio/syu080 25261464 10.1093/sysbio/syu080 PMC4265144

[CR79] Dolezel J, Greilhuber J, Suda J (2007) Estimation of nuclear DNA content in plants using flow cytometry. Nat Protoc 2(9):2233–2244. 10.1038/nprot.2007.310 10.1038/nprot.2007.310 17853881

[CR21] Fan H, Ives AR, Surget-Groba Y, Cannon CH (2015) An assembly and alignment-free method of phylogeny reconstruction from next-generation sequencing data. BMC Genomics 16(1):522. 10.1186/s12864-015-1647-5 26169061 10.1186/s12864-015-1647-5 PMC4501066

[CR22] Feschotte C, Jiang N, Wessler SR (2002) Plant transposable elements: where genetics meets genomics. Nat Rev Genet 3:329–341. 10.1038/nrg793 11988759 10.1038/nrg793

[CR23] Finot VL, Baeza CM, Ruiz E, Toro O, Carrasco P (2018a) Towards an integrative taxonomy of the genus *Alstroemeria* (Alstroemeriaceae) in Chile: a comprehensive review. Selected studies in biodiversity, chapter 12. 10.5772/intechopen.71823

[CR77] Finot VL, Baeza C, Muñoz- Schick, Ruiz E, Espejo J, Alarcón D, Carrasco P, Novoa P, Eyzaguirre MT (2018b) Guía de campo de Alstroemerias chilenas (Ed) Corporación Chilena de la Madera, Concepción, Chile, 292 pp

[CR25] Garrido-Ramos MA (2015) Satellite DNA in plants: more than just rubbish. Cytogenet Genome Res 146:153–170. 10.1159/000437008 26202574 10.1159/000437008

[CR26] Garrido-Ramos MA (2017) Satellite DNA: an evolving topic. Genes 8:230–271. 10.3390/genes8090230 28926993 10.3390/genes8090230 PMC5615363

[CR27] Garrido-Ramos MA (2021) The genomic of plant satellite DNA. Prog Mol Subcell Biol 60:103–143. 10.1007/978-3-030-74889-0_5 34386874 10.1007/978-3-030-74889-0_5

[CR28] Girardi LB, Peiter MX, Pimenta BD, Bruning J, Rodrigues AS, Kirchner JH (2017) Crescimento e desenvolvimento da *Alstroemeria* x hybrida quando submetida a diferentes capacidades de retenção de vaso. Rev Bras Agric Irrig 11:1191–1200. 10.7127/rbai.v11n100561

[CR30] Guerra M (2000) Patterns of heterochromatin distribution in plant chromosomes. Genet Mol Biol 23:1029–1041. 10.1590/S1415-47572000000400049

[CR31] Guerra M (1988) Introdução à Citogenética Geral. Guanabara Koogan, Rio de Janeiro, RJ. 143 pp

[CR32] Hemleben V, Kovarik A, TorresRuiz RA, Volkov RA, Beridze T (2007) Plant highly repeated satellite DNA: molecular evolution, distribution and use for identification of hybrids. Syst Biodivers 5:277–289. 10.1017/S147720000700240X

[CR82] Katoh K, Standley DM (2013) MAFFT multiple sequence alignment software version 7: improvements in performance and usability. Mol Biol Evol. 10.1093/molbev/mst010 10.1093/molbev/mst010 PMC3603318 23329690

[CR81] Kearse M, Moir R, Wilson A, Stones-Havas S, Cheung M, Sturrock S, Buxton S, Cooper A, Markowitz S, Duran C, Thierer T, Ashton B, Meintjes P, Drummond A (2012) Geneious Basic: an integrated and extendable desktop software platform for the organization and analysis of sequence data. Bioinformatics 28(12):1647–1649. 10.1093/bioinformatics/bts199 10.1093/bioinformatics/bts199 PMC3371832 22543367

[CR34] Kelly LJ, Renny-Byfield S, Pellicer J, Macas J, Novák P, Neumann P, Lysak MA, Day PD, Berger M, Fay MF et al (2015) Analysis of the giant genomes of *Fritillaria* (Liliaceae) indicates that a lack of DNA removal characterizes extreme expansions in genome size. New Phytol 208:596–607. 10.1111/nph.13471 26061193 10.1111/nph.13471 PMC4744688

[CR36] Leitch IJ, Leitch AR (2013) Genome size diversity and evolution in land plants. In: Greilhuber J, Dolezel J, Wendel J (eds) Plant genome diversity volume 2. Springer, Vienna. 10.1007/978-3-7091-1160-4_19

[CR78] Loureiro J, Rodriguez E, Doležel J, Santos C (2007) Two new nuclear isolation buffers for plant DNA flow cytometry: a test with 37 species. Ann Botany 100:875–888. 10.1093/aob/mcm152 10.1093/aob/mcm152 PMC2749623 17684025

[CR37] Macas J, Novák P, Pellicer J, Čížková J, Koblížková A, Neumann P, Fuková I, Dolezel J, Kelly LJ, Leitch IJ (2015) In depth characterization of repetitive DNA in 23 plant genomes reveals sources of genome size variation in the legume tribe *Fabeae*. PLoS ONE 10:1–23. 10.1371/journal.pone.0143424 10.1371/journal.pone.0143424 PMC4659654 26606051

[CR38] McCann J, Macas J, Novák P, Stuessy TF, Villaseñor JL, Weiss-Schneeweiss H (2020) Differential genome size and repetitive DNA evolution in diploid species of *Melampodium* sect. *Melampodium *(Asteraceae). Front Plant Sci 11:362. 10.3389/fpls.2020.00362 32296454 10.3389/fpls.2020.00362 PMC7136903

[CR40] Montenegro C, Ibiapino A, Nascimento T, da Costa AF, Brasileiro-Vidal AC, Pedrosa-Harand A (2025) Cytogenomic and phylogenomic evidence for new infrageneric relationships in *Macroptilium* (Benth.) beans. Ann Bot. 10.1093/aob/mcaf151 10.1093/aob/mcaf151 PMC12823238 40668962

[CR41] Muñoz-López M, García-Pérez JL (2010) DNA transposons: nature and applications in genomics. Curr Genomics 11:115–128. 10.2174/138920210790886871 20885819 10.2174/138920210790886871 PMC2874221

[CR42] Nascimento J, Nollet F, Guerra M, Felix LP (2025b) Morphological and karyotypic characterization of *Alstroemeria urubiciensis* (Alstroemeriaceae): a new species from Santa Catarina, Brazil. Phytotaxa 687:278–286. 10.11646/phytotaxa.687.2.6

[CR43] Nascimento J, Sader M, Ribeiro T, Pedrosa-Harand A (2025a) Influence of Ty3/gypsy and Ty1/copia LTR retrotransposons on the large genomes of Alstroemeriaceae: genome landscape of *Bomarea edulis* (Tussac) Herb. Protoplasma 262:881–894. 10.1007/s00709-025-02036-2 39883160 10.1007/s00709-025-02036-2

[CR44] Neumann P, Novák P, Hoštáková N, Macas J (2019) Systematic survey of plant LTR retrotransposons elucidates phylogenetic relationships of their polyprotein domains and provides a reference for element classification. Mob DNA 10:1 30622655 10.1186/s13100-018-0144-1 PMC6317226

[CR45] Novák P, Neumann P, Pech J, Steinhaisl J, Macas J (2013) RepeatExplorer: a Galaxy- based web server for genome-wide characterization of eukaryotic repetitive elements from nextgeneration sequence reads. Bioinformatics 29:792–793. 10.1093/bioinformatics/btt054 23376349 10.1093/bioinformatics/btt054

[CR46] Novák P, Ávila LR, Koblížková A, Vrbová I, Neumann P, Macas J (2017) TAREAN: a computational tool for identification and characterization of satellite DNA from unassembled short reads. Nucleic Acids Res 45:111. 10.1093/nar/gkx257 10.1093/nar/gkx257 PMC5499541 28402514

[CR47] Novák P, Guignard MS, Neumann P, Kelly LJ, Mli-narec J, Koblizkova A, Dodsworth S, Kovarık A, Pellicer J, Wang W, Macas J, Leitch IJ, Leitch AR (2020a) Repeat-sequence turnover shifts fundamentally in species with large genomes. Nat Plants 6:1325–1329. 10.1038/s41477-020-00785-x 33077876 10.1038/s41477-020-00785-x

[CR48] Novák P, Neumann P, Macas J (2020b) Global analysis of repetitive DNA from unassembled sequence reads using RepeatExplorer2. Nat Protoc 15:3745–3776. 10.1038/s41596-020-0400-y 33097925 10.1038/s41596-020-0400-y

[CR84] Pedrosa A, Sandal N, Stougaard J, Schweizer D, Bachmair A (2002) Chromosomal map of the model legume Lotus japonicus. Genetics 161:1661–1672. 10.1093/genetics/161.4.1661 10.1093/genetics/161.4.1661 PMC1462223 12196409

[CR50] Pellicer J, Hidalgo O, Dodsworth S, Leitch IJ (2018) Genome size diversity and its impact on the evolution of land plants. Genes 9:88. 10.3390/genes9020088 29443885 10.3390/genes9020088 PMC5852584

[CR51] Peška V, Mandáková T, Ihradská V, Fajkus J (2019) Comparative dissection of three giant genomes: *Allium cepa*, *Allium sativum*, and *Allium ursinum*. Int J Mol Sci 20:733. 10.3390/ijms20030733 30744119 10.3390/ijms20030733 PMC6387171

[CR83] Price MN, Dehal PS, Arkin AP (2009) FastTree: computing large minimum evolution trees with profiles instead of a distance matrix. Mol Biol Evol. 10.1093/molbev/msp077 10.1093/molbev/msp077 PMC2693737 19377059

[CR52] Rao SR, Trivedi S, Emmanuel D, Merita K, Hynniewta M (2010) DNA repetitive sequences-types, distribution and function: a review. J Cell Mol Biol. 7(2) & 8(1):1–11

[CR54] Ribeiro T, Vasconcelos E, dos Santos KGB, Vaio M, Brasileiro-Vidal AC, Pedrosa-Harand A (2020) Diversity of repetitive sequences within compact genomes of *Phaseolus* L. beans and allied genera *Cajanus* L. and *Vigna* Savi. Chromosome Res 28:139–153. 10.1007/s10577-019-09618-w 31734754 10.1007/s10577-019-09618-w

[CR55] Ribeiro T, Nascimento J, Santos A, Félix LP, Guerra M (2021a) Origin and evolution of highly polymorphic rDNA sites in *Alstroemeria longistaminea* (Alstroemeriaceae) and related species. Genome 64:833–845. 10.1139/gen-2020-0159 33852822 10.1139/gen-2020-0159

[CR56] Ribeiro T, Vaio M, Felix LP, Guerra M (2021b) Satellite DNA probes of *Alstroemeria longistaminea* (Alstroemeriaceae) paint the heterochromatin and the B chromosome, reveal a G-like banding pattern, and point to a strong structural karyotype conservation. Protoplasma 259:413–426. 10.1007/s00709-021-01681-7 34148192 10.1007/s00709-021-01681-7

[CR80] Ruiz-Ruano FJ, López-León MD, Cabrero J, Camacho JPM (2016) High-throughput analysis of the satellitome illuminates satellite DNA evolution. Sci Rep 6:28333. 10.1038/srep28333 10.1038/srep28333 PMC4935994 27385065

[CR57] Sader M, Vaio M, Cauz-Santos LA, Dornelas MC, Vieira MLC, Melo N, Pedrosa-Harand A (2021) Large vs small genomes in *Passiflora*: the influence of the mobilome and the satellitome. Planta 253:86. 10.1007/s00425-021-03598-0 33792791 10.1007/s00425-021-03598-0

[CR58] Saksouk N, Simboeck E, Déjardin J (2015) Constitutive heterochromatin formation and transcription in mammals. Epigenetics Chromatin 8:3. 10.1186/1756-8935-8-3 25788984 10.1186/1756-8935-8-3 PMC4363358

[CR60] Sanso AM (1996) El género *Alstroemeria* (Alstroemeriaceae) em Argentina. Darwiniana 34:349–382. 10.14522/darwiniana.2014.341-4.424

[CR61] Sanso AM (2002) Chromosome studies in Andean taxa of *Alstroemeria* (Alstroemeriaceae). Bot J Linn Soc 138:451–459. 10.1046/j.1095-8339.2002.00019.x

[CR62] Sanso AM, Hunziker JH (1998) Karyological studies in *Alstroemeria* and *Bomarea* (Alstroemeriaceae). Hereditas 129:67–74. 10.1111/j.1601-5223.1998.t01-1-00067.x

[CR63] Schnable PS, Ware D, Fulton RS, Stein JC, Wei F, Pasternak S et al (2009) The B73 maize genome: complexity, diversity, and dynamics. Science 326:1112–1115. 10.1126/science.1178534 19965430 10.1126/science.1178534

[CR64] Soltis PS, Soltis DE (2020) Plant genomes: markers of evolutionary history and drivers of evolutionary change. Plants People Planet 3:74–82. 10.1002/ppp3.10159

[CR65] Thakur J, Packiaraj J, Steven H (2021) Sequence, chromatin and evolution of satellite DNA. Int J Mol Sci 22:4309. 10.3390/ijms22094309 33919233 10.3390/ijms22094309 PMC8122249

[CR67] Usai G, Mascagni F, Natali L et al (2017) Comparative genome-wide analysis of repetitive DNA in the genus *Populus* L. Tree Genet Genomes 13:96. 10.1007/s11295-017-1181-5

[CR68] Vaio M, Nascimento J, Mendes S, Ibiapino A, Felix LP, Gardner A, Emshwiller E, Fiaschi P, Guerra M (2018) Multiple karyotype changes distinguish two closely related species of *Oxalis* (*O. psoraleoides* and *O. rhombeo-ovata*) and suggest an artificial grouping of section Polymorphae (Oxalidaceae). Bot J Linn Soc 188:269–280. 10.1093/botlinnean/boy054

[CR69] Van-lume B, Mata-Sucre Y, Báez M, Ribeiro T, Huettel B, Gagnon E, Souza G (2019) Evolutionary convergence or homology? Comparative cytogenomics of *Caesalpinia* group species (Leguminosae) reveals diversification in the pericentromeric heterochromatic composition. Planta 250:2173–2186. 10.1007/s00425-019-03287-z 31696317 10.1007/s00425-019-03287-z

[CR71] Weising K, Nybom H, Pfenninger M, Wolff K, Kahl G (2005) DNA fingerprinting in plants: principles, methods, and applications. CRC Press. 10.1093/aob/mcj057

[CR72] Wood TE, Takebayashi N, Barker MS, Mayrose I, Greenspoon PB, Rieseberg LH (2009) The frequency of polyploid speciation in vascular plants. Proc Natl Acad Sci USA 106:13875–13879. 10.1073/pnas.0811575106 19667210 10.1073/pnas.0811575106 PMC2728988

[CR73] Xie DF, Tan JB, Yu Y, Gui LJ, Su DM, Zhou SD, He XJ (2020) Insights into phylogeny, age and evolution of *Allium* (Amaryllidaceae) based on the whole plastome sequences. Ann Bot 1(7):1039–1055. 10.1093/aob/mcaa024 10.1093/aob/mcaa024 PMC7262478 32239179

[CR75] Zanela L (2009) Dissertação: Caracterização cariotípica de quatro espécies brasileiras de *Alstroemeria* (Alstroemeriaceae) com as técnicas de FISH, CMA, DAPI e AgNOR. Dissertation. Instituto agronômico de Campinas (IAC), Campinas, Brasil, 79 pp

