# Supplementary material for: Conservation of giant genome structure in Brazilian and Chilean species of the genus Alstroemeria L. (Alstroemeriaceae), despite dynamism in satellite repeats
Source: Planta. 2026 Feb 6;263(3):69. doi: 10.1007/s00425-026-04933-z (PMC12876544; doi:10.1007/s00425-026-04933-z)
Supplement: Supplementary file 1 — Supplementary file1 (DOCX 380 KB) [file 425_2026_4933_MOESM1_ESM.docx]

**SUPPLEMENTARY MATERIAL**


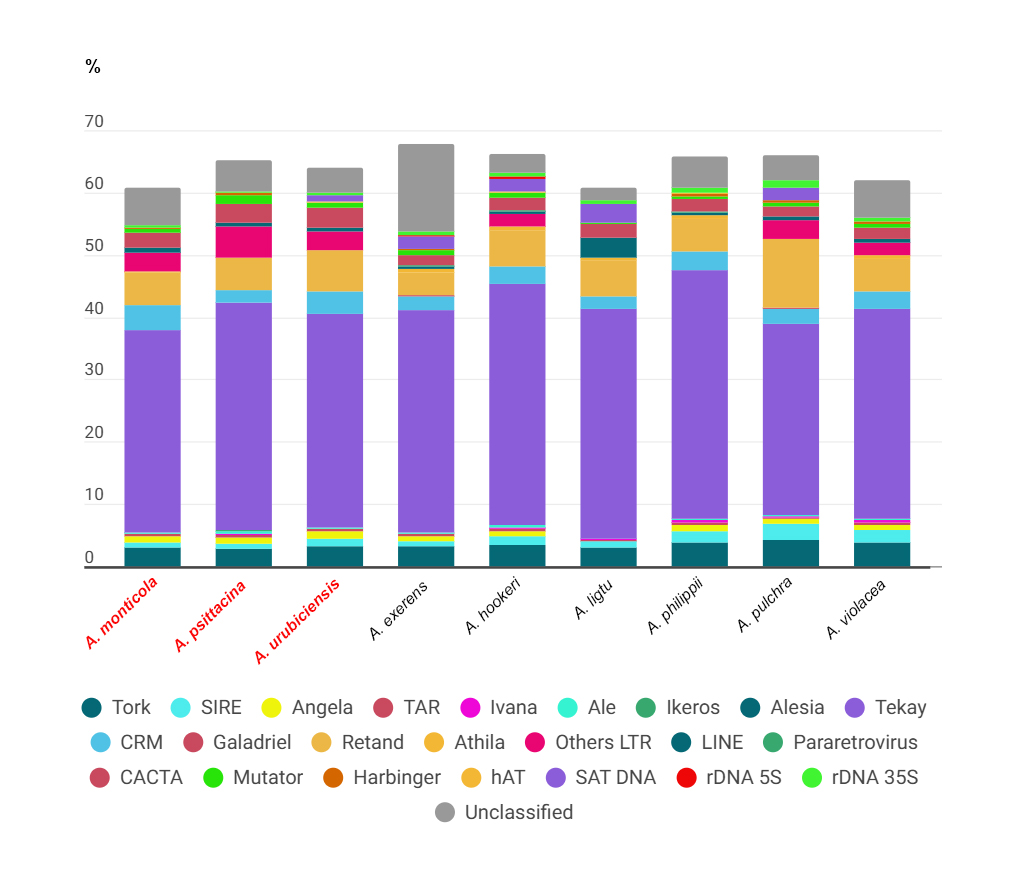


**Supplementary Figure 1.** Proportions of repeats in *Alstroemeria* species after individual analysis with RepeatExplorer. The first four species are Brazilian.


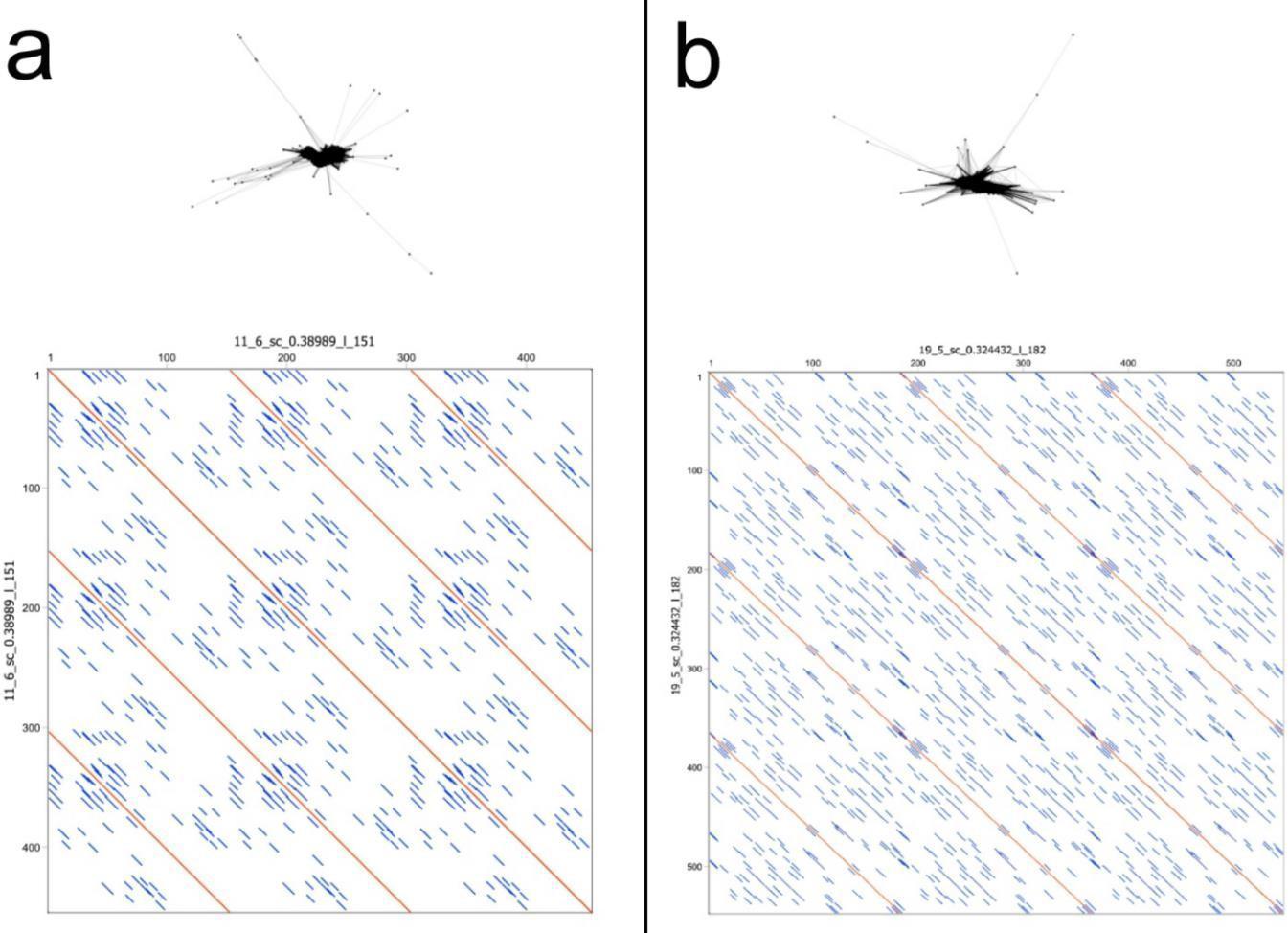


**Supplementary Figure 2.** TAREAN graph and dotplot of new *Alstroemeria* satellite DNAs selected for chromosomal mapping. In a, AhoSAT2-361 of *A. hookeri* and in b, AliSAT1-285 of *A. ligtu.*


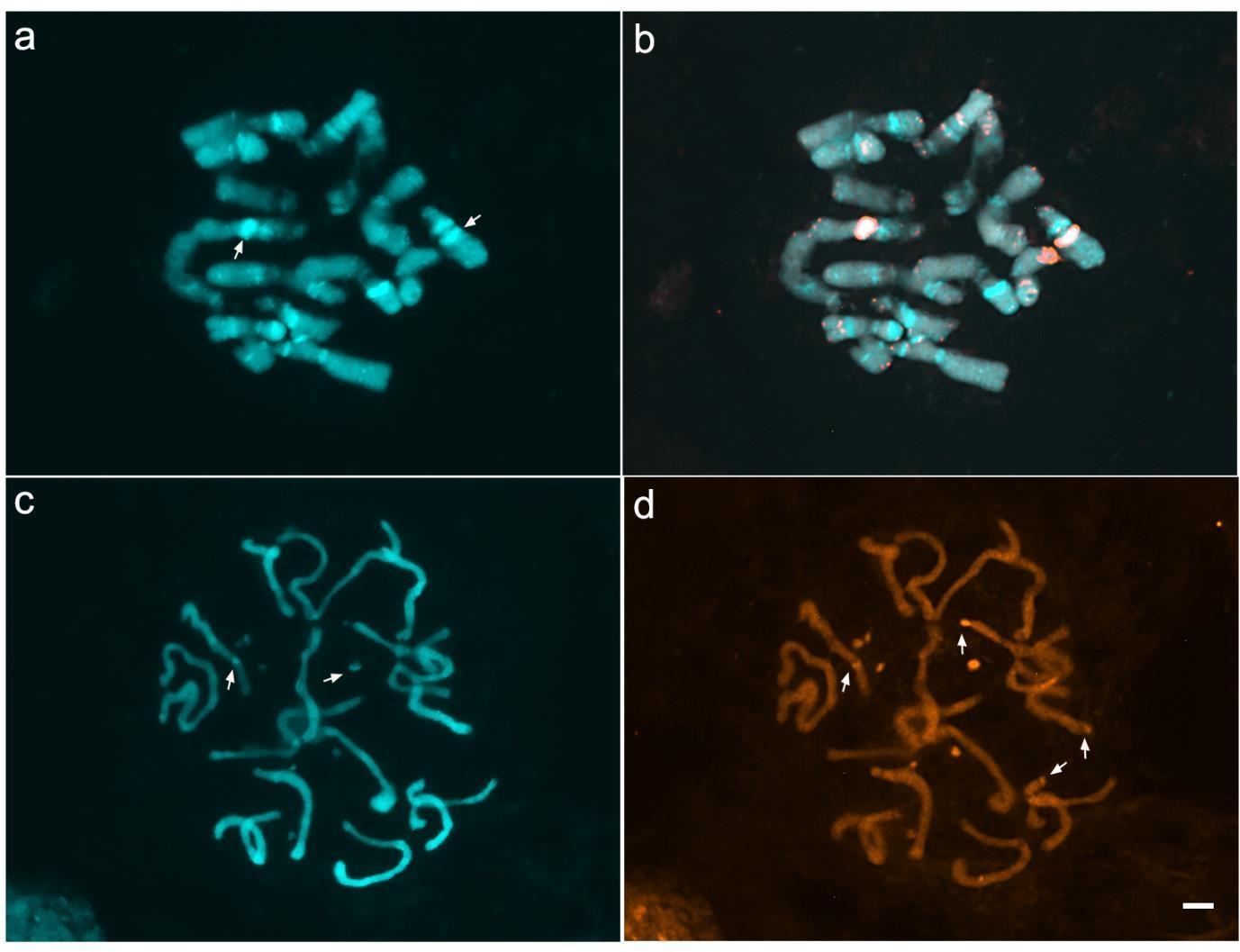


**Supplementary Figure 3.** Fluorescent *in situ* hybridization of satDNAs in Chilean *Alstroemeria* species. a-b) AhoSAT2-361 in *A. ligtu*. c-d) AliSAT1-285 in *A. hookeri*. White arrows in a and c show DAPI blocks co-located with satellites. White arrows in d show weak satellite signals. Bar = 10µ.

**Supplementary Table 1.** Genomic proportions (%) of repetitive sequences identified in species of *Alstroemeria* after comparative RepeatExplorer analysis.

|  | ***A.***  ***exerens*** | ***A.***  ***hookeri*** | ***A.***  ***ligtu*** | ***A. longistaminea*** | ***A. monticola*** | ***A.***  ***philippii*** | ***A.***  ***psittacina*** | ***A.***  ***pulchra*** | ***A.***  ***violacea*** | ***A.***  ***urubiciensis*** |
| --- | --- | --- | --- | --- | --- | --- | --- | --- | --- | --- |
| **Genome size (1C)** | - | 26.00 pg –  25.42 Gb | 34.70 pg –  33.94 Gb | 26.37 pg – 25.8  Gb | 26.55 pg – 25,97 Gb* | 20.75 pg –  20.29 Gb | 24.95 pg –  24.40 Gb | 20.05 pg –  19.61 GB | 29.15 – 28.51  Gb* | 26.48 pg –  25,90 Gb* |
| **HKL (μm)** | **-** | **-** | - | **-** | **-** | **-** |  | **-** | 118.25 μm | - |
| **Comparative clustering reads** | 622.730 | 607.382 | 608.328 | 649.280 | 557.148 | 606.124 | 724.394 | 539.346 | 611.498 | 558.864 |
| **Coverage** | - | 0.004x | 0.003x | 0.004x | 0.003x | 0.004x | 0.004x | 0.004x | 0.003x | 0.003x |
| **Repetitive elements** |  |  |  |  | **Genome proportion (%)** | |  |  |  |  |
| **Class I** |  |  |  |  |  |  |  |  |  |  |
| **LTR-retrotransposon** | | | | | | | | | | |
| **Ty1/copia** |  |  |  |  |  |  |  |  |  |  |
| **Tork** | 3.60 | 3.25 | 3.21 | 2.96 | 3.23 | 3.63 | 3.01 | 4.10 | 3.72 | 3.27 |
| **SIRE** | 0.61 | 0.88 | 0.81 | 0.78 | 0.70 | 1.12 | 0.68 | 1.34 | 1.14 | 0.68 |
| **Angela** | 0.84 | 0.84 | 0.59 | 1.29 | 1.08 | 0.97 | 1.05 | 0.65 | 0.91 | 1.04 |
| **TAR** | 0.22 | 0.21 | 0.19 | 0.16 | 0.21 | 0.20 | 0.2 | 0.18 | 0.20 | 0.21 |
| **Ivana** | 0.43 | 0.76 | 0.62 | 0.29 | 0.34 | 0.85 | 0.35 | 1.09 | 1.0 | 0.38 |
| **Ale** | 0.20 | 0.22 | 0.15 | 0.17 | 0.18 | 0.22 | 0.21 | 0.22 | 0.21 | 0.20 |
| **Ty3/gypsy** |  |  |  |  |  |  |  |  |  |  |
| ***Chromovirus*** |  |  |  |  |  |  |  |  |  |  |
| **Tekay** | 34.14 | 33.55 | 31.40 | 35.60 | 35.64 | 33.14 | 37.00 | 27.22 | 30.21 | 36.07 |

| **CRM** | 2.31 | 2.33 | 2.19 | 2.05 | 1.83 | 2.24 | 1.84 | 2.24 | 2.40 | 1.79 |
| --- | --- | --- | --- | --- | --- | --- | --- | --- | --- | --- |
| **Galadriel** | 0.07 | 0.07 | 0.07 | 0.06 | 0.07 | 0.07 | 0.08 | 0.08 | 0.1 | 0.07 |
| **Non-chromovirus** |  |  |  |  |  |  |  |  |  |  |
| **Retand** | 3.77 | 4.85 | 4.73 | 4.46 | 4.43 | 4.35 | 3.97 | 9.89 | 5.03 | 4.65 |
| **Athila** | 0.05 | 0.03 | 0.04 | 0.03 | 0.03 | 0.03 | 0.04 | 0.03 | 0.04 | 0.04 |
| **Others LTRs** | 5.44 | 4.83 | 4.53 | 5.42 | 5.21 | 5.30 | 5.55 | 3.87 | 5.21 | 5.10 |
| **Non-LTR** |  |  |  |  |  |  |  |  |  |  |
| **LINE** | 0.30 | 0.26 | 0.35 | 0.38 | 0.41 | 0.30 | 0.36 | 0.36 | 0.30 | 0.42 |
| **Pararetrovirus** | 0.03 | 0.03 | 0.02 | 0.06 | 0.06 | 0.08 | 0.05 | 0.01 | 0.05 | 0.05 |
| **Class II** |  |  |  |  |  |  |  |  |  |  |
| **CACTA** | 1.81 | 1.84 | 1.90 | 2.53 | 2.37 | 1.76 | 2.44 | 1.51 | 1.34 | 2.43 |
| **Mutator** | 0.47 | 0.41 | 0.37 | 0.47 | 0.52 | 0.49 | 0.55 | 0.45 | 0.48 | 0.52 |
| **Harbinger** | 0.25 | 0.29 | 0.22 | 0.29 | 0.29 | 0.26 | 0.25 | 0.30 | 0.31 | 0.25 |
| **hAT** | 0.09 | 0.07 | 0.08 | 0.08 | 0.1 | 0.09 | 0.1 | 0.10 | 0.12 | 0.10 |
| **SatDNA** | 2.44 | 3.06 | 5.9 | 1.72 | 1.50 | 1.33 | 1.51 | 1.67 | 1.40 | 1.83 |
| **rDNA** |  |  |  |  |  |  |  |  |  |  |
| **5S** | 0.09 | 0.42 | 0.09 | 0.06 | 0.05 | 0.02 | 0.05 | 0.03 | 0.02 | 0.06 |
| **35S** | 0.92 | 0.70 | 3.20 | 0.42 | 0.30 | 0.89 | 0.19 | 1.27 | 0.63 | 0.45 |
| **Unclassifield repeats** | 6.8 | 5.62 | 5.72 | 5.25 | 7.20 | 6.30 | 5.73 | 6.51 | 6.11 | 6.00 |
| **Total** | 64.88 | 64.52 | 66.38 | 64.52 | 65.75 | 63.64 | 65.21 | 63.11 | 61.15 | 65.61 |

References of the 2C value in picograms: Buitendijk et al. 1997, Nascimento et al. 2021a.

*Genomes sizes estimated in this present work.

**Supplementary Table 2.** Consensus sequences by TAREAN and primers used for amplification of satellites of *A. ligtu* and *A. hookeri*.

| **> AliSAT1-285** |  |
| --- | --- |
| **Consensus** | CCAATGACAACAACGACAACGCATGGCGATGCCAAGTACCACGCCGGACAGTGCCAAGCGCAATGCCGGACGGTACCAAGC ACCGTCACGGGTGGTGGGGCCAAATTCCGTCGCACGTGGGGTGGGGCCAAACAACGCCGCGCGCGGTGGTCGGGGGGCC AAATTCCGCCGCACGCGGTGGTCGGGCCAAACAAGCACCATGCCGGAAGGTGCAAAGTGCCGTGCCGGAGGGGGCCAAGC  ACTATGCCGGAAGGTGCAAAGCACCGCCGAGCGGCGCGCGGGGC |
| **Primer sequence (5’**🡪**3’)** |  |
| AliSAT1 F | GGCCCAATGACAACAACGAC |
| AliSAt1 R | CTTTGCACCTTCCGGCATAG |
| **> AhoSAT2-361** |  |
| **Consensus** | CGTTCGCGTCGAAAAGATCTACGTTTTGACAGGTCATTCGCCCAATTCCAACGGTCGGATCGAAAGTTAGGCCGTTTCCGGG AAACCCTAGAGATTTGGGTGGTAAAATGAACCCTTGACCCTGAAATCGAAACCCTGGACCCTAAACTCGTAACCCAGAACACT AAACCATGAACCCTAAACCCTAAACCTTAAACTCGAAACCCTAACCCTAAACTCGTGACCCGAAACCCTAAAATCGTGACCCT AAACCCTAAACTCGTGACCCCAAACCCTAAAGTCGTGACCCTAAACCCTAAACTCGGAACCCTAAACCCTGAACTCGTGACCC  TAAACCCTAAACCCTAAACCCTGTATCG |
| **Primer sequence (5’**🡪**3’)** |  |
| AhoSAT2 F | CGGATCGAAAGTTATGGCCG |
| AhoSAT2 R | CCGTTGGAATTGGGCGAATG |

**Supplementary Table 3.** Proportion of the main satellite DNAs identified in *Alstroemeria* species by comparative analyses.

|  |  | CG  content (%) |  |  |  |  | Abundance (%) | |  |  |  |  |
| --- | --- | --- | --- | --- | --- | --- | --- | --- | --- | --- | --- | --- |
| SatDNA | Monomer size (bp) |  | Brazilian species | |  |  |  |  | Chilean species | |  |  |
|  |  |  | *A.*  *urubuciensis* | *A.*  *longistaminea* | *A.*  *monticola* | *A.*  *psittacina* | *A.*  *exerens* | *A.*  *hookeri* |  | *A.*  *philippii* | *A.*  *pulchra* |  |
|  |  |  |  |  |  |  |  |  | *A. ligtu* |  |  | *A.violacea* |
| AliSAT1 | 774 | 43.8% | 0.045441 | 0.03931 | 0.033 | 0.041342 | 0.058209 | 0.060432 | 4.668378 | 0.081152 | 0.052819 | 0.053116 |
| AurSAT3 | 226 | 50.0% | 0.450741 | 0.593174 | 0.550582 | 0.382175 | 0.01146 | 0.030136 | 0.049368 | 0.065609 | 0.021592 | 0.07055 |
| AviSAT2 | 103 | 70.9% | 0 | 0 | 0 | 0 | 0.186088 | 0.304563 | 0.305614 | 0.196827 | 0.703244 | 0.208716 |
| Other 1 | 1614 | 33.3% | 0.189105 | 0.172641 | 0.184057 | 0.181962 | 0.128788 | 0.185943 | 0.113156 | 0.191264 | 0.127587 | 0.215722 |
| AviSAT3 | 6124 | 40.0% | 0.074104 | 0.137343 | 0.018882 | 0.136333 | 0.518244 | 0.343354 | 0.109394 | 0.149052 | 0.153996 | 0.140447 |
| ApuSAT6 | 3596 | 42.1% | 0.160267 | 0.151623 | 0.145234 | 0.171038 | 0.209007 | 0.166227 | 0.199825 | 0.160669 | 0.143825 | 0.175641 |
| AexSAT5 | 138 | 47.8% | 0.158345 | 0.170716 | 0.148763 | 0.162051 | 0.144432 | 0.150518 | 0.118171 | 0.154779 | 0.132226 | 0.174989 |
| AliSAT8 | 318 | 43.1% | 0.232624 | 0.176813 | 0.260467 | 0.242109 | 0.105868 | 0.094575 | 0.079146 | 0.103895 | 0.074054 | 0.118615 |
| AexSAT1 | 22 | 59.1% | 0 | 0 | 0.000353 | 0 | 0.830573 | 0.691357 | 0 | 0 | 0 | 0.000652 |
| AhoSAT5 | 1129 | 34.0% | 0.363878 | 0.145526 | 0.018 | 0.033461 | 0.003274 | 0.284846 | 0.004232 | 0.016689 | 0.074054 | 0.013523 |
| AexSAT6 | 163 | 52.8% | 0.040023 | 0.033373 | 0.040588 | 0.052957 | 0.097864 | 0.079667 | 0.076169 | 0.082952 | 0.063883 | 0.090264 |
| Other 2 | 885 | 38.95 | 0.076726 | 0.073164 | 0.070234 | 0.064848 | 0.048386 | 0.060432 | 0.035106 | 0.05252 | 0.04122 | 0.048717 |
| AhoSAT2 | 361 | 47.6% | 0.00035 | 0 | 0 | 0.000138 | 0.000546 | 0.502849 | 0 | 0.000164 | 0.000178 | 0 |
| Other 3 | 212 | 46.75 | 0.022895 | 0.021821 | 0.019941 | 0.020602 | 0.047113 | 0.038952 | 0.037457 | 0.044339 | 0.031584 | 0.047087 |
| AhoSAT10 | 154 | 48.7% | 0 | 0.000321 | 0 | 0 | 0.030924 | 0.036868 | 0.091528 | 0.012762 | 0.044075 | 0.010916 |
| Other 4 | 832 | 24.9% | 0.012234 | 0.007381 | 0.013412 | 0.017698 | 0.017099 | 0.029975 | 0.011911 | 0.012762 | 0.006424 | 0.013849 |
